# A mathematical model of the metastatic bottleneck predicts patient outcome and response to cancer treatment

**DOI:** 10.1371/journal.pcbi.1008056

**Published:** 2020-10-02

**Authors:** Ewa Szczurek, Tyll Krüger, Barbara Klink, Niko Beerenwinkel

**Affiliations:** 1 Faculty of Mathematics, Informatics and Mechanics, University of Warsaw, Warsaw, Poland; 2 Faculty of Electronics, Wrocław University of Science and Technology, Wrocław, Poland; 3 Institute for Clinical Genetics, Faculty of Medicine Carl Gustav Carus, Technische Universität Dresden, Dresden, Germany; 4 National Center of Genetics, Laboratoir national de santé, Dudelange, Luxembourg; 5 Department of Biosystems Science and Engineering, ETH Zurich, Basel, Switzerland; 6 SIB Swiss Institute of Bioinformatics, Basel, Switzerland; Max-Planck-Institute for Evolutionary Biology, GERMANY

## Abstract

Metastases are the main reason for cancer-related deaths. Initiation of metastases, where newly seeded tumor cells expand into colonies, presents a tremendous bottleneck to metastasis formation. Despite its importance, a quantitative description of metastasis initiation and its clinical implications is lacking. Here, we set theoretical grounds for the metastatic bottleneck with a simple stochastic model. The model assumes that the proliferation-to-death rate ratio for the initiating metastatic cells increases when they are surrounded by more of their kind. For a total of 159,191 patients across 13 cancer types, we found that a single cell has an extremely low median probability of successful seeding of the order of 10^−8^. With increasing colony size, a sharp transition from very unlikely to very likely successful metastasis initiation occurs. The median metastatic bottleneck, defined as the critical colony size that marks this transition, was between 10 and 21 cells. We derived the probability of metastasis occurrence and patient outcome based on primary tumor size at diagnosis and tumor type. The model predicts that the efficacy of patient treatment depends on the primary tumor size but even more so on the severity of the metastatic bottleneck, which is estimated to largely vary between patients. We find that medical interventions aiming at tightening the bottleneck, such as immunotherapy, can be much more efficient than therapies that decrease overall tumor burden, such as chemotherapy.

## Author summary

We propose a stochastic model of metastasis formation, encompassing the release of cells from a growing tumor, their settlement at a distant site and their impact on patient outcome. We put forward a theory of the extreme bottleneck that this process encounters at the settlement step. We derive equations for several clinically relevant quantities as functions of primary tumor size for patients who underwent surgery: the probability of metastasis occurrence and of metastasis detection, the risk of death due to cancer, and the survival time. The model is fit to epidemiological data for 13 cancer types, and used to predict the impact of different treatment options on patient outcome.

## Introduction

**Fig 1:**
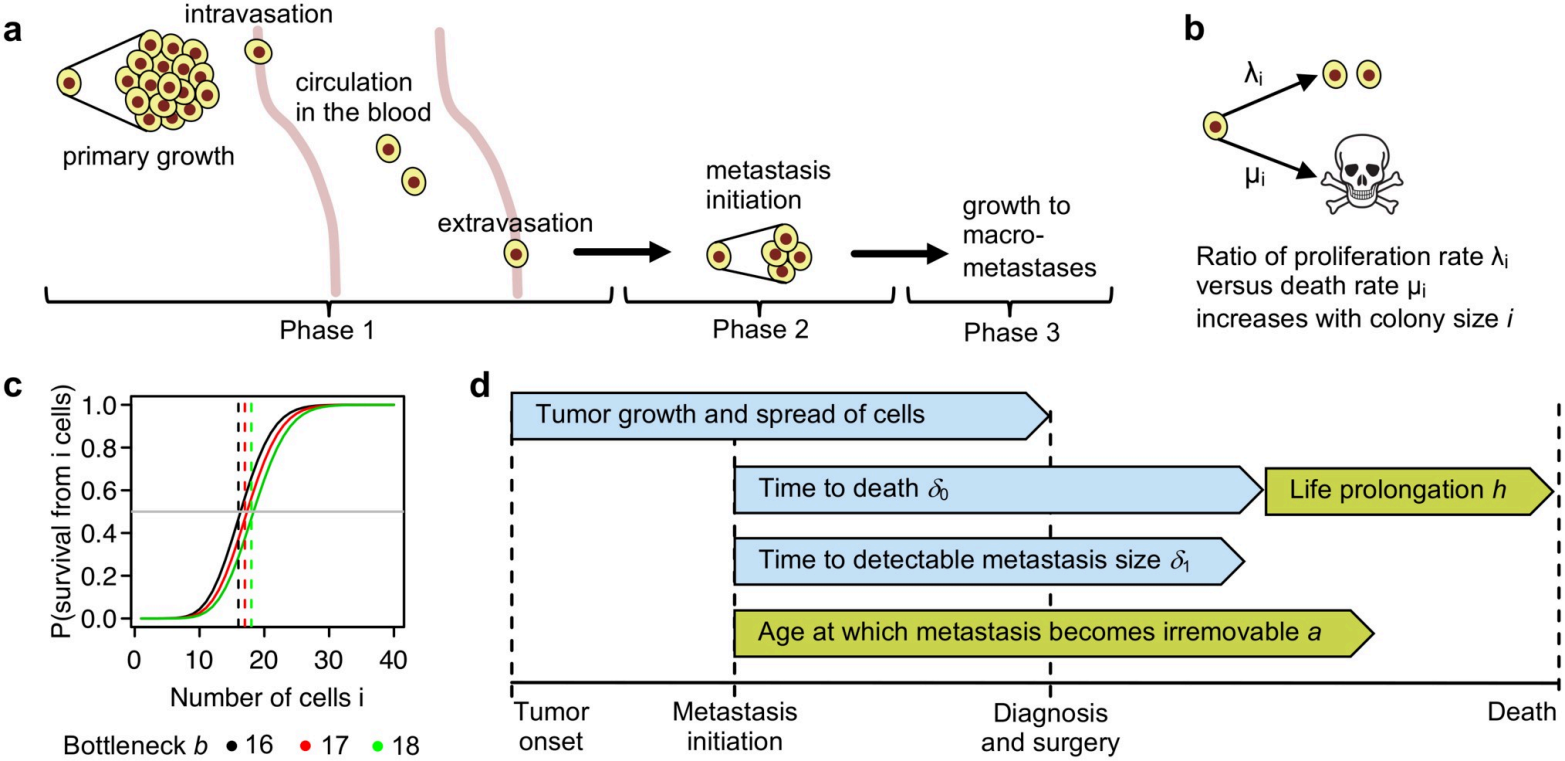
Modeling metastasis formation process and its bottleneck. **a** Three phases of metastasis formation. In phase 1, tumor cells are constantly released into the blood stream. Given that larger tumors can release more cells over time, they have a higher chance to develop metastases. Thus, a dependency of metastasis probability and patient outcome on tumor size is expected. The bottleneck is the metastasis initiation in phase 2. **b** Stochastic birth-and-death process of metastasis initiation. For each cell in the forming colony, the ratio of its proliferation rate λ_*i*_ to its death rate *μ*_*i*_ depends on the total number of cells *i* with a proportionality constant $\frac{1}{b}$. **c** The bottleneck severity *b* is the critical colony size for metastasis initiation. The probability of colony survival starting from *i* cells is lower than 0.5 (gray horizontal line) when *i* < *b* and larger than 0.5 when *i* > *b*, and increases rapidly when *i* crosses *b*. *b* = 17, red, is the most common median bottleneck estimated for cancers we analyzed (Fig 4d). **d** Model parameters on a timescale. From tumor onset to diagnosis, the tumor grows and releases cells. Once a metastasis is successfully initiated, it takes a cancer-specific average number of years *δ*_0_ until it becomes lethal. The initiated metastases grow on average *δ*_1_ years to become large enough to be detectable by screening. With a certain probability, decreasing with metastasis age, and parametrized by the expected age *a* when metastases become irremovable, treatment may remove metastasis. Also due to treatment, time to death is prolonged by a constant *h*.

Metastases are responsible for 90% of deaths from cancer [1, 2]. The formation of metastases is a multi-step process, in which tumor cells spread from the primary site and colonize distant organs [3]. It can be divided into three phases (Fig 1a). The first phase consists of tumor cell entry into the vascular system (intravasation), transport in the blood, and exit from the vascular system to a secondary organ (extravasation). This step may or may not be preceded by the acquisition of genetic or epigenetic alterations in the primary tumor [4–6]. Experimental data suggests that the tumor cells that are shed from the primary site are already equipped with metastatic abilities [7–9]. Recent findings support that metastatic tumor cell dissemination begins in early [10, 11], rather than late stage of the disease. The first phase of metastasis formation is highly efficient, as the released tumor cells deal remarkably well with the obstacles of delivery to distant organs and their infiltration [4, 5, 12–14]. In contrast, the second phase—metastasis initiation—is extremely inefficient [4, 6, 15, 16]. Relative to the huge numbers of cells that disseminate during the long period of primary tumor growth, only very few of them successfully form distant metastases [12, 17]. This bottleneck is commonly understood as the lack of compatibility of the seeded tumor cells with the soil they encounter in the affected organ [4, 18, 19]. In mice models, the metastatic seeding potential was observed to increase with the size of tumor cell clumps [20], which was recently confirmed for human circulating tumor cell clusters [21]. In the last phase of metastasis formation, successfully initiated colonies form micrometastases and, subsequently, clinically detectable macrometastases [6].

The metastatic process has been previously modeled using various mathematical approaches. In their pioneering work, Liotta and colleagues proposed a series of mathematical models of the metastatic process in mice [22–24]. They also first described that larger implanted tumor clumps have a larger chance to seed metastases [20]. That finding was not taken into account in theoretical models developed since then. Hartung *et al*. [25] modeled primary and metastatic growth in mice, assuming that metastatic cells are constantly released from the surface of the growing tumor with a given rate. In the branching process model of metastasis formation by Avanzini and Antal, the metastatic lesions are initiated at a rate which depends on the size of the primary tumor [26]. Michor *et al*. [27] proposed a constant population size Moran process model of evolutionary emergence of pro-metastatic mutations in the primary tumor. This model was later extended to account for expanding tumor size and the cell release probability [28], and was put into a clinical context [29, 30]. Haeno *et al*. [30] fitted parameters of this model to primary and metastatic cell count measurements determined post-mortem in pancreatic cancer patients. Benzekry *et al*. [31] modeled the non-linear dependence of the probability of metastatic relapse on primary tumor size, finding that between-patient variability of intrinsic metastatic potential is a key parameter, and suggesting that tumor size alone has a limited utility as a predictor of metastatic recurrence. None of the previous studies accounted for the metastatic bottleneck explicitly. Haeno *et al*. [29, 30] implicitly identified the rate-limiting step with the emergence of pro-metastatic mutations in the primary tumor. Cisneros and Newman [32] proposed a stochastic process model of metastasis colonization depending on a constant corresponding to proliferation-to-death ratio. In contrast to the here presented model, that model assumed, rather than predicted, a switching behavior of the probability that the metastatic colony survives, did not account for bottleneck variability, and was not fit to patient data (see S1 Text for a detailed comparison of the models).

The mechanism behind metastasis initiation and the bottleneck that is encountered at this phase remain poorly understood [6]. A quantitative characterization of the metastatic bottleneck may facilitate more efficient cancer treatment strategies, as it presents a natural point of attack of this fatal disease. Our metastasis bottleneck model is motivated by size-dependent seeding strategies seen in ecology. For example, plants produce either large numbers of small seeds or smaller numbers of large seeds, which have higher chances of survival in stressful conditions [33]. Similarly, *Leptothorax* ants form larger colonies to survive harsh winter weather, which are divided into smaller nests during the summer [34]. The general Allee effect was described, amounting to a positive correlation between population size and individual fitness [35]. The Allee principle was previously suggested to govern cancer cell population growth kinetics at low densities [36]. The Allee effect is likely to also play a role in metastasis initiation, because out of an extremely large number of attempts only very few metastatic seedings are successful [6], and the success probability depends on the colony size, as determined experimentally [20, 21]. Once accompanied by more of their own kind, the tumor cells should benefit from a more friendly, tumor-like microenvironment and are less vulnerable to restrictive conditions at the secondary site. Accordingly, in the model, the bottleneck of metastasis formation results from a stochastic process of metastatic colony initiation (phase 2) where the ratio of proliferation to death rates of cells in the colony increases with its size (Fig 1b). With increase of the colony size this stochastic process of colony initialization undergoes a rapid but smooth transition from a subcritical to a supercritical process when passing a critical colony size (Fig 1c).

To validate our model and hypotheses and to be able to make predictions based on epidemiological data, we make the following assumptions. First, since larger tumors release more cells in total over their lifetime, they should have higher chances to seed metastases and therefore result in poorer prognosis. Tumor size itself is indeed an important factor of the standard tumor/node/metastasis (TNM) cancer staging system [37], which is important for prognosis and treatment decision making. Studies analyzing the dependence of metastasis incidence on tumor size date back to the 1980’s [38]. Importantly, breast cancer patient survival was observed to become independent of tumor size upon occurrence of metastases [39]. This observation further supports the causative, and not only correlative, dependence of metastatic incidence on tumor size. Similarly to previous studies, we assume exponential primary tumor growth [29, 30], that tumors release metastatic cells from their surface [25], and that surgery removes the source of metastatic seeding [31]. Based on these assumptions and on our stochastic model of the metastatic bottleneck, we derive analytical expressions for metastasis probability, metastasis detection and cancer death probabilities, and any quantile (for example, median) time to death as functions of primary tumor size at diagnosis. Our expressions closely fit to and are predictive of epidemiological data for thirteen different cancer types. Our results, predictions, and estimated parameters quantitatively characterize the metastatic bottleneck and indicate that it is a promising therapeutic target.

## Methods

We introduce a mathematical model of the tumor growth, extravasation, and intravasation, the metastatic bottleneck, as well as the metastasis probability and clinical outcome (Fig 1).

### Mathematical model of tumor growth, extravasation, and intravasation

To describe the first and efficient phase of metastasis formation, we assume that as the primary tumor grows, it continues to release cells from its surface (Fig 1a). Primary tumor growth is modeled as an exponentially increasing spherical volume with the doubling time set to constants measured for different cancers (S1 Table). The per-cell per-year release rate is fixed to match experimental data, showing that a tumor of one gram contains about 10^9^ cells [40] and that such a tumor sheds around 1.5 × 10^5^ cells per day [22]. The extravasation probability is fixed to 0.8, based on experimental observations in mice [12].

### Mathematical model of the metastatic bottleneck

To model the second phase of metastasis initiation, we rely on the observations that (*i*) it is extremely inefficient [4, 6, 15, 16] and (*ii*) metastatic propensity increases with colony size [20, 21]. Seemingly in contrast to the second assumption, in large enough metastatic colonies with high enough density, cells undergo necrosis due to local overcrowding, and their proliferation rate is expected to decrease and the death rate is expected to increase. In the case of metastasis initiation, however, the situation is the opposite. At the alien secondary site, tumor cells in the starting colony lack their usual surrounding in the primary tissue and are attacked by the immune system. The larger the colony, the less vulnerable are its cells to these restrictive conditions. Accordingly, our model is an inhomogeneous birth-death process, where the ratio of proliferation (λ_*i*_) to death (*μ*_*i*_) rates of each individual cell depends on the colony size *i* with a proportionality constant 1/*b*

$$\begin{array}{c} \frac{\lambda_{i}}{\mu_{i}}=\frac{i}{b}. \end{array}$$ (1)

We refer to the parameter *b* as the bottleneck severity (Fig 1b). The larger the severity *b*, the stronger the metastatic bottleneck. Indeed, the larger *b*, the smaller the proliferation to death rate ratio. Let

$$\begin{array}{c} p_{i}=\frac{\lambda_{i}}{\lambda_{i}+\mu_{i}} \end{array}$$ (2)

denote the jump probability from state *i* to *i* + 1, and 1 − *p*_*i*_ the probability for the jump from *i* into *i* − 1. The jump probability fully describes the stochastic dynamics of the population. For large jump probability *p*_*i*_ the colony size will likely increase in the next step, while for small *p*_*i*_ it will likely decrease. The critical colony size is reached when this probability passes 1/2. Inserting the rates in relation described by Eq (1) into Eq (2), we find that the critical size is equal to ⌈*b*⌉. This branching process has a single absorbing state *i* = 0, corresponding to colony extinction.

We refer to the probability *s*_1_ of never reaching the absorbing state starting from a single initial cell as the metastasis success probability, given by *s*_1_ = exp(−*b*) (S1 Text). Hence, the metastasis success probability exponentially approaches 0 with rate equal to the bottleneck severity *b*. To make the dependence of *s*_1_ on the bottleneck severity *b* explicit, in further considerations we will use the notation *s*_1_(*b*). The probability of colony survival (not going extinct), starting from any number of cells *i* > 1 reads *s*_*i*_(*b*) = *F*_*Pois*_(*i* − 1; *b*), where *F*_*Pois*_(*i*; *b*) denotes a cumulative Poisson distribution function with parameter *b* (S1 Text). Thus, as the colony size passes the critical number of *i* = ⌈*b*⌉ cells, the probability of survival from *i* cells *s*_*i*_(*b*) passes 0.5, rapidly switching from small to large values (Fig 1c).

It is of course not possible that the proliferation to death ratio goes to infinity with increasing colony size. In S1 Text we show that a model assuming a truncation to a constant ratio after the colony grows large, would yield only negligibly different probabilities of colony survival. In addition, we present theoretical extension of the model to a more general case, where $\frac{\lambda_{i}}{\mu_{i}}=(\frac{i}{b}{)}^{\alpha }$, with addition of parameter *α*. We discuss a possible scenario where the cells on the colony surface are more vulnerable to alien microenvironment, leading to value *α* = 1/3.

Let *t* be the time from from the onset of the tumor to the time of diagnosis. At this time point, the primary tumor has released a very large number *N*(*t*) of cells that can be regarded as independent trials to initiate metastases, each with very small success probability *s*_1_(*b*). The number of metastases is thus Poisson distributed with rate *N*(*t*) ⋅ *s*_1_(*b*), and the probability of having at least one successfully initiated metastasis at time *t* is

$$\begin{array}{c} M\left(t;b\right)=1-\text{exp}[-s_{1}\left(b\right)\cdot N(t)]. \end{array}$$ (3)

### Modeling patient outcome

To model post-surgical patient outcome, we make the following assumptions (Fig 1d). We assume that complete removal of the primary tumor by surgery eliminates the source of new metastatic seeding, that treatment may remove the metastases and prolong lifespan, and that the first successful metastasis results in patient death. We ignore potential secondary seeding from (micro-)metastases, which may be present at the time of the surgery. We assume that such secondary seeding does not affect the patient outcome, which instead depends on the first metastasis that was not removed by treatment. Once metastasis initiation succeeds in one site of the body, it takes an average time *δ*_0_ for metastasis to become lethal. An average time *δ*_1_ is required for the metastases to grow large enough to be detectable at diagnosis. Removal of metastases by systemic therapy is less probable as they grow, and the time when the metastases become irremovable is exponentially distributed with expectation *a*. Finally, we consider that treatment prolongs survival of patients on average by *h* years. Thus, the average time to death from the first acquired metastasis, which was later not removed by treatment is *δ*_0_ + *h*. The parameters *δ*_0_, *δ*_1_, *a*, *h* are deterministic. In contrast, the bottleneck severity *b* may vary between metastatic colonies and individual patients. We model this variability by assuming that the bottleneck severity is log-normally distributed with parameters *μ* and *σ*. The six quantities *δ*_0_, *δ*_1_, *a*, *h*, *μ*, and *σ* differ for each cancer type. They are the free parameters of the model and the derived analytical expressions for (*i*) the probability of cancer death, (*ii*) the probability of metastasis detection, (*iii*) the quantile times to cancer death for all patients, and (*iv*) the quantile times to cancer death for the subset of patients with detected metastases.

Below, we introduce the equations describing the dependency of important clinical variables on tumor size at diagnosis. Their full derivations are presented in S1 Text.

#### Post-surgical cancer death probability for patients diagnosed with given tumor size

Let *t*(*d*) denote the time that elapsed from the onset of the tumor to the time point when it is diagnosed with diameter *d*. *t*(*d*) is easily computed with the assumptions of exponential growth of the primary tumor and its spherical shape (S1 Text). We first observe that patients presenting with a tumor of size *d* should not have developed metastases before *t*(*d*) − *δ*_0_. This accounts for the trivial fact that the diagnosed patient is alive at the time of diagnosis. Furthermore, post-surgical therapy, most commonly chemotherapy, may eradicate some of metastases that could be life-threatening without treatment. Parameter *a* corresponds to the expected age of metastases when they become irremovable by treatment. For a given bottleneck severity *b*, let *M*(*t*; *a*, *b*, *δ*_0_) denote the metastasis probability at time point *t* conditioned on the event that up to time point *t*(*d*) − *δ*_0_ no successful metastasis has been created and that successful metastases were not removed by treatment after the diagnosis (derived in S1 Text):

$$\begin{array}{c} M\left(t;a,b,\delta_{0}\right)=1-\text{exp}[-s_{1}\left(b\right)N\left(t;a,\delta_{0}\right)], \end{array}$$ (4)

where *N*(*t*; *a*, *δ*_0_) is the number of such metastatic seeding attempts that happened after *t*(*d*) − *δ*_0_, but also early enough to become irremovable by treatment.

Finally, we account for the fact that the bottleneck may vary between metastatic colonies and individual patients. Such variability is expected due to multiple biological factors that may affect the bottleneck, such as differences in genetic makeup and metastatic potential between tumor cells, and in the microenvironment between the various organs within the body where they try to seed, as well as in immune system strength and metabolic rate between patients. We model this variability of the bottleneck severity *b* by assuming that it is a random variable with log-normal density *f*(*b*; *μ*, *σ*) with the cancer-specific parameters: location *μ* and scale *σ*. The cancer death probability at time *t* is thus obtained by marginalizing over the distribution of the bottleneck severity

$$\begin{array}{c} M\left(t;a,\delta_{0},\mu ,\sigma \right)=\int_{0}^{\infty }M\left(t;a,b,\delta_{0}\right)f\left(b;\mu ,\sigma \right)db. \end{array}$$ (5)

Finally, to compute the dependence of cancer death probability on tumor size at diagnosis, we calculate

$$\begin{array}{c} M\left(d;a,\delta_{0},\mu ,\sigma \right)=\int_{0}^{\infty }M\left[t\left(d\right);a,b,\delta_{0}\right]f\left(b;\mu ,\sigma \right)db. \end{array}$$ (6)

#### Metastasis detection probability for patients diagnosed with given tumor size

We assume that each metastasis needs a mean time *δ*_1_ till it reaches a detectable size. Let *t*_1_(*d*) = *t*(*d*) − *δ*_1_ and *δ*_1_ < *δ*_0_. We obtain metastasis detection probability as a function of tumor diameter

$$\begin{array}{c} M(d;\delta_{0},\delta_{1},\mu ,\sigma )=\int_{0}^{\infty }M\left[t_{1}\left(d\right);b,\delta_{0}\right]f\left(b;\mu ,\sigma \right)db, \end{array}$$ (7)

where *M*[*t*; *b*, *δ*_0_] is the metastasis probability at time point *t*, conditioned on the fact that up to time point *t*(*d*) − *δ*_0_, where *t*(*d*) is the time of diagnosis, no successful metastasis has been created (S1 Text). Thus, the metastasis detection probability depends on the parameters *δ*_0_, *δ*_1_, *μ*, and *σ*. Metastasis detection does not depend on the parameter *a*, since we assume the screening for metastases occurs at diagnosis, before any treatment that could remove metastases.

#### Quantile time to death from patients diagnosed with given tumor size

We consider the *q*-th quantile time to death of cancer for patients who (a) had a surgery following the diagnosis with diameter *d*, and (b) will indeed die of cancer. With *x*_*q*_ being the root of the equation (S1 Text)

$$\begin{array}{c} \int_{0}^{\infty }\frac{M(x_{q};a,b,\delta_{0})}{M[t\left(d\right);a,b,\delta_{0}]}f\left(b;\mu ,\sigma \right)db=q,\;t\left(d\right)>x_{q}\geq t_{0}\left(d\right). \end{array}$$ (8)

the quantile time to death is given by

$$\begin{array}{c} Q(d,q;a,\delta_{0},h,\mu ,\sigma )=x_{q}+\delta_{0}-t(d)+h, \end{array}$$ (9)

where *h* is an additional treatment-related parameter that accounts for the increase of patient survival due to therapy after detection of the tumor. Thus, the expression for the quantile time to death depends on the cancer-specific parameters *a*, *δ*_0_, *h*, *μ*, and *σ*, where the dependence on *a*, *μ*, and *σ* is introduced via *x*_*q*_.

#### Quantile time to death from cancer for the subset of patients with detected metastases at diagnosis with given tumor size

For the subset of patients with detected metastases, the quantile time to death should be computed conditioning on the fact that the metastases originated before *t*_1_(*d*) = *t*(*d*) − *δ*_1_ (otherwise they would not have been detectable) and after *t*_0_(*d*) = *t*(*d*) − *δ*_0_ (otherwise the patient would have died prior to diagnosis). Reasoning analogously to above, we solve

$$\begin{array}{c} \int_{0}^{\infty }\frac{M(x_{q}^{\prime };a,b,\delta_{0})}{M[t_{1}\left(d\right);a,b,\delta_{0}]}f\left(b;\mu ,\sigma \right)db=q,\;t_{1}\left(d\right)>x_{q}^{\prime }\geq t_{0}\left(d\right) \end{array}$$ (10)

for $x_{q}^{\prime }$, and the quantile time to death for patients with metastases detectable at diagnosis is then given by

$$\begin{array}{c} Q^{\prime }(d,q;a,\delta_{0},h,\mu ,\sigma )=x_{q}^{\prime }+\delta_{0}-t(d)+h. \end{array}$$ (11)

### Predicting impact of treatment decisions

To estimate the impact of surgery delay *δ* for a fixed bottleneck severity *b* and given tumor size *d* at diagnosis, we compute the difference of metastasis probability (equivalently, probabilities of cancer death; given by Eq 4), with and without the delay

$$\begin{array}{c} \Delta \left(d;\delta ,a,b,\delta_{0}\right)=M\left[t\left(d\right)+\delta ;\;a,\;b,\;\delta_{0}\right]-M\left[t\left(d\right);\;a,\;b,\;\delta_{0}\right]. \end{array}$$ (12)

Similarly, the marginal impact of surgery delay for a given tumor size at diagnosis, marginalized over possible bottleneck severities, is computed using using Eq 5 as the difference

$$\begin{array}{c} \Delta \left(d;\delta ,a,\delta_{0},\mu ,\sigma \right)=M\left[t\left(d\right)+\delta ;\;a,\;\delta_{0},\;\mu ,\;\sigma \right]-M\left[t\left(d\right);\;a,\;\delta_{0},\;\mu ,\;\sigma \right]. \end{array}$$ (13)

Next, we model the increase of chemotherapy efficiency as an increase of the average metastasis age when the metastasis becomes irremovable by treatment, i.e., by multiplying the parameter *a* by a factor *c*_1_ > 1. To quantify the impact of increased chemotherapy efficiency for a fixed bottleneck severity *b*, we compare the cancer death probabilities with and without the increase,

$$\begin{array}{c} \Delta \left(d;c_{1},a,b,\delta_{0}\right)=M\left[t\left(d\right);\;c_{1}\cdot a,\;b,\;\delta_{0}\right]-M\left[t\left(d\right);\;a,\;b,\;\delta_{0}\right]. \end{array}$$ (14)

Similarly, the marginal impact of increased chemotherapy efficiency is evaluated as

$$\begin{array}{c} \Delta \left(d;c_{1},a,\delta_{0},\mu ,\sigma \right)=M\left[t\left(d\right);\;c_{1}\cdot a,\;\delta_{0},\;\mu ,\;\sigma \right]-M\left[t\left(d\right);\;a,\;\delta_{0},\;\mu ,\;\sigma \right]. \end{array}$$ (15)

Finally, we predict the impact of the increase of the metastasis bottleneck severity by a factor *c*_2_ > 1. Such an increase would correspond to the mechanism of action of vaccines, where we would be able to strengthen the defense of the immune system against the initiation of the metastatic colonies. For a fixed initial bottleneck severity *b*, the impact is evaluated as the difference between the cancer death probability for increased *b* and for *b* unchanged

$$\begin{array}{c} \Delta \left(d;c_{2},a,b,\delta_{0}\right)=M\left[t\left(d\right);\;a,\;c_{2}\cdot b,\;\delta_{0}\right]-M\left[t\left(d\right);\;a,\;b,\;\delta_{0}\right]. \end{array}$$ (16)

To compute the marginal impact of increased bottleneck severity, we evaluate the difference of the cancer death probability for increased median of the bottleneck distribution and for the median unchanged

$$\begin{array}{c} \Delta \left(d;c_{2},a,\delta_{0},\mu ,\sigma \right)=M\left[t\left(d\right);\;a,\;\delta_{0},\;\log\left(c_{2}\right)+\mu ,\;\sigma \right]-M\left[t\left(d\right);\;a,\;\delta_{0},\;\mu ,\;\sigma \right]. \end{array}$$ (17)

Since the median of the log-normal distribution is given by exp(*μ*), the new distribution of the bottleneck parameter has location parameter log(*c*_2_) + *μ*.

### Code availability

The code allowing full reproducibility of all presented results is freely available at https://github.com/EwaSzczurek/MetastaticBottleneck.

## Results

### Dependence of clinical outcome on tumor size as observed in epidemiological data

We systematically analyzed the records of patients selected from the SEER database [41]. The data included fourteen cancer types from eleven primary sites, namely ductal and lobular breast, ovarian, endometrial, esophageal, gastric, colon and mucionous colon, rectal, pancreatic, non-small cell lung, head and neck, renal, and bladder cancer. We selected a total of 159,191 patients with a single primary tumor that was surgically removed without prior treatment and where primary tumor diameter was measured (for details about SEER data and patient selection see S1 Text and S2 Table).

**Fig 2:**
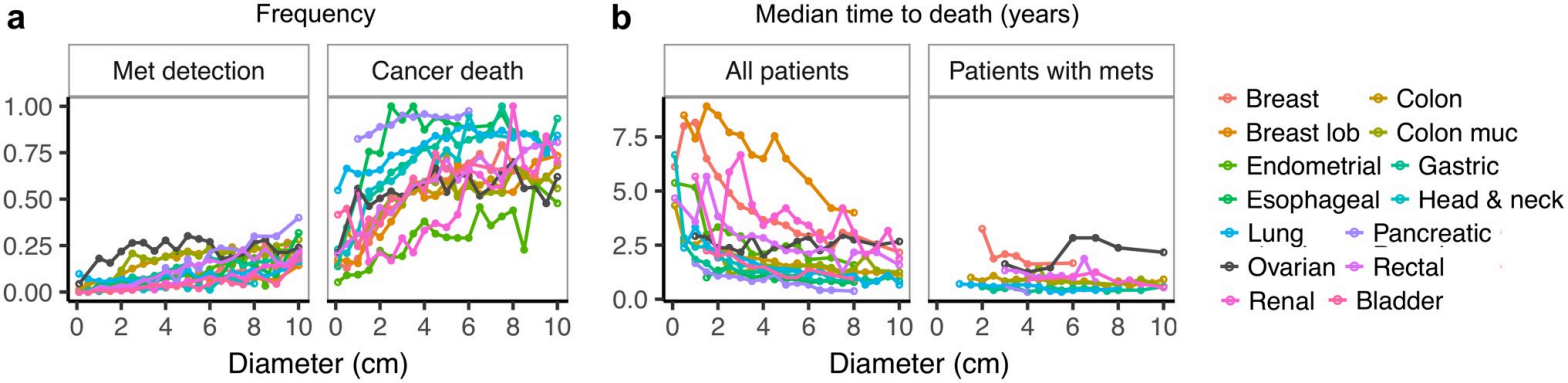
Epidemiological data from the SEER database [41] for different cancer types (colors). **a** The probability of metastasis detection (left) and the probability of cancer death (right) increase with tumor size at diagnosis. Only ovarian cancer data (black) do not follow these monotonic trends. **b** Median time to death for all patients (left) decreases with tumor size at diagnosis, again except for ovarian cancer (black). For the subset of patients who had metastases detected at diagnosis (right), the median time to death is generally much shorter and is not tumor-size dependent.

We first investigated how clinical outcome depends on tumor size. For all cancer types except for ovarian cancer, we found that the frequency of metastasis detection and of cancer death significantly increase with tumor diameter (Fig 2a), while median time to death decreases (Fig 2b left). S3 Table reports significance test results obtained from two-sided Mann-Kendall trend tests. Ovarian cancer data does not follow this trend, possibly because ovarian carcinoma has a unique and very efficient way to rapidly spread within the peritoneal cavity, which differs markedly from the classical pattern of hematogenous dissemination [42]. This deviating behavior further supports the connection between tumor size and metastatic probability via hematogenous tumor cell dissemination. The difference in trends is even more pronounced when an average over all non-ovarian cancer cell types is compared against ovarian cancer (S1 Fig). The patients with metastases detectable at diagnosis die of cancer much earlier than patients without detected metastasis, and their median time to death does not depend on primary tumor diameter (Fig 2b right). This finding is in agreement with previous observations made for breast cancer [39].

### Model fitting and validation

**Fig 3:**
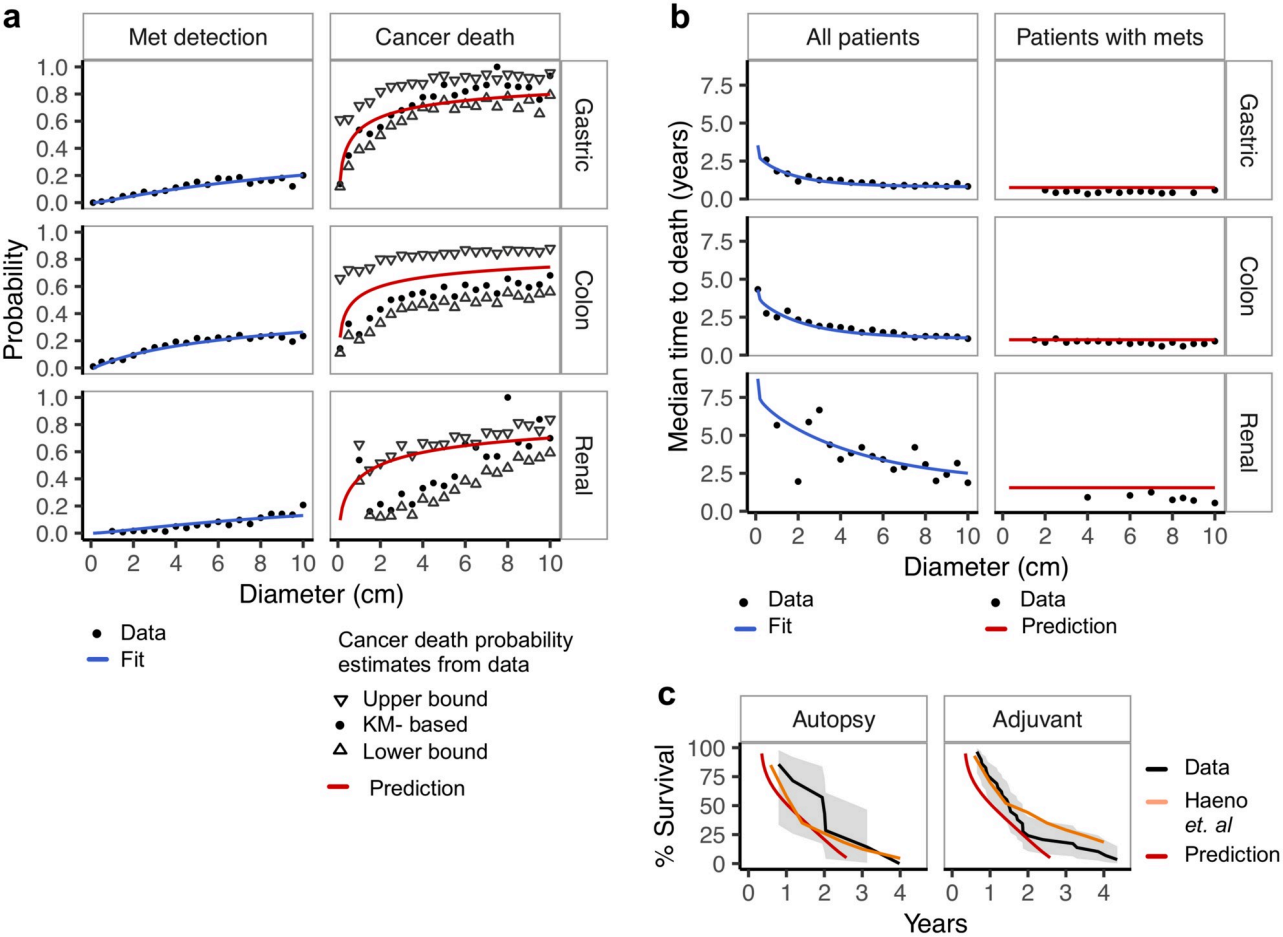
Model fit and validation. **a,b** Fit and validation of our model on the SEER data, exemplary for three cancers, for which we obtained the best, intermediate, and worse fit, respectively, and for which validation data was available (see S2 Fig for remaining ten cancers). Black points represent data records, blue lines show fitted, and red lines predicted curves. **a** Metastasis detection probability (left panel) is compared to cancer death probability (right). For cancer death probability, up and down-oriented triangles present the upper and lower estimates of that variable from the data, respectively, while the black dots represent a Kaplan-Meier (KM) derived estimate (S1 Text). The predictions stay within the range of the estimators. **b** The median time to death data for all patients, (left panel) was used for model fitting and is tumor-size dependent. For a validation cohort of patients with metastases detected at diagnosis, the model correctly predicts a much shorter median time to death and that this time is almost constant across tumor diameters (right). **c** Independent validation on survival data for two pancreatic cancer cohorts: Autopsy and Adjuvant, analyzed by Haeno *et al*. [30] (black, with gray confidence bands). We predict their survival function using our pancreas cancer model, fit solely to SEER data, and accessing only information about tumor diameters of patients in the cohorts. For both cohorts, our predictions (red) are as close to the data as predictions obtained from the the Haeno *et al*. model (orange).

To estimate the model parameters for each cancer type, we fitted the expressions for metastasis detection (Eq 7; Fig 3a and S2a Fig, blue curves) and quantile times to death (Eq 9; Fig 3b and S2b Fig, blue curves) for all patients and eleven different quantiles to the SEER data (see Methods for equations and S1 Text for model fitting and validation procedure). These expressions together depend on all six free parameters of our model. The ovarian cancer data was excluded from this analysis because it did not show dependence of patient outcome on tumor size in the epidemiological data, which is in contrast to our model assumptions (Fig 2). The model fits the data extremely well. Fits to other quantile times to death data are similar to the fits to the median (S3 Fig).

With all parameters estimated, we validated the model by comparing its predictions of cancer death probability (predicted using Eq 6) to the corresponding data in the SEER database (Fig 3a and S2a Fig, red curves). Here, the validation concerns the correctness of Eq 6, as the model was trained fitting Eqs 7 and 9, without using the information about the fraction of patients who eventually died of cancer. Cancer death probability, however, is difficult to estimate from epidemiological data due to deaths for other reasons. To account for those deaths, we reported three different estimators for cancer death probability. The upper-bound estimator treats all deaths as cancer death, the lower-bound estimator treats all patients who died from other reasons than cancer as alive, and the Kaplan-Meier estimator treats other deaths as censored (S1 Text). We find that the model predictions are consistent with observed cancer death frequencies, such that for most cancer types our predictions are very close to one of its estimators. Cancer death probability is modeled as the probability of having metastasis that were not removed by treatment after diagnosis. This accounts also for undetectable metastases, and thus the predicted cancer death probability is larger than metastasis detection probability, in agreement with the data.

Furthermore, we predicted the time to death for the group of patients who had metastases detected at the time of diagnosis (using Eq 11). For these patients, the metastasis must have originated so early that it had enough time to grow to a detectable size (Methods). The model accurately predicts the very short median time to cancer death for patients with detected metastases and that it does not depend on tumor diameter (Fig 3b and S2b Fig, red curves).

Additionally, we validated our model on independent survival data from two pancreatic cancer cohorts, named Autopsy and Adjuvant [30]. Using our expressions that we fitted to pancreatic cancer data from the SEER, we very closely predict the survival functions of these two independent cohorts using only their primary tumor diameters (Fig 3c; S1 Text). Our predictions are as close to the data as those obtained from the model of Haeno *et al*. [30], which, however, was fitted to the Autopsy cohort itself, having access not only to tumor diameters and clinical information, but also to counts and sizes of metastatic lesions determined at autopsy.

### Characterization of cancer types with model parameter values

**Fig 4:**
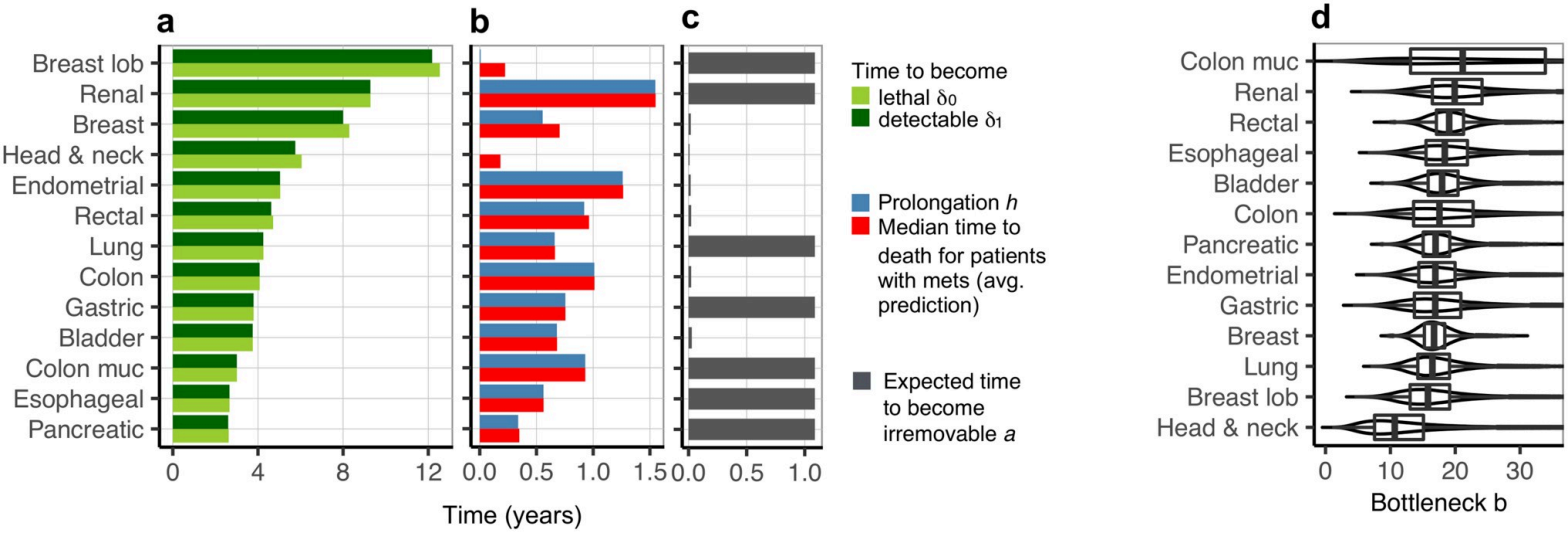
Estimated model parameters for thirteen cancers. **a** The estimated time for metastases to become lethal, *δ*_0_ (light green) and the time for metastases to become detectable, *δ*_1_ (dark green), are shorter for more aggressive cancers. **b** The estimated life prolongation due to treatment *h* (blue) is very close to the predicted median time to death for patients with metastases detected at diagnosis, averaged over tumor diameters (red; compare Fig 3b). **c** The expected time of metastases to become irremovable by treatment, *a*, was estimated to be the upper bound of around 1 year, for seven cancers. For the remaining six cancers, *a* is on the order of weeks and the removal probability decreases much faster as a function of metastasis age. **d** Box plots showing 25th, 50th (median), and 75th percentiles (vertical bars) and 1.5 interquartile ranges (horizontal line ends) of the bottleneck severity *b* (x-axis) for all cancers, ordered by the median severity (y-axis). The median bottleneck severities range from 10 to 21, with the most common median severity equal 17 cells (the median bottleneck severity was 17 for four cancers, for three it was 18, and for two it was 16). As expected, the variances of the bottleneck severity distributions are large, indicating strong variability of the bottleneck both within and between patients.

The estimated model parameters allowed ranking cancer types by their severity and susceptibility to treatment (Fig 4a). The estimated time intervals from the formation of a successful metastatic colony to the patient’s death, *δ*_0_, are different for different cancer types and range from 2.6 years for the deadliest up to 12.5 years for the least aggressive cancers. For all cancers, the estimated parameters *δ*_1_, interpreted as the time during which the metastases grow detectable, are very close to *δ*_0_. This has important implications to model predictions. According to the model assumptions, patients die on average *δ*_0_ (the time for a metastasis to become lethal) plus *h* (life prolongation due to treatment) years after the acquisition of the first metastasis, which was later not removed by systemic therapy, such as chemotherapy. First, the fact that *δ*_1_ is close to *δ*_0_ indicates that patients with detectable metastases are already at a very late stage of the disease and without treatment would have died at a time point close to diagnosis and metastasis detection. Second, the predicted median time to death for those patients is determined by treatment induced life prolongation *h* (Fig 4b), in good agreement with the data (compare Fig 3b). For all cancer types, the estimated prolongation *h* is much shorter than the intervals *δ*_0_ and *δ*_1_, with the largest values of around 1.5 years. The times for the metastases to become lethal (*δ*_0_) and detectable (*δ*_1_), as well as life prolongation *h* tend to be shorter for cancer types that are known to be more aggressive, such as pancreatic or esophageal carcinomas. The expected age *a* when the metastases become irremovable was either estimated around 1 year (its upper bound; S1 Text) or only several weeks (Fig 4c).

The estimated distribution of the bottleneck severity *b* provides new insights into the number of tumor cells critical for successful metastasis initiation. The distribution is similar across all analyzed cancers, with one outlier, namely colon mucinous cancer (Fig 4d). The most common median bottleneck is 17 cells, corresponding to a metastatic success probability of only *s*_1_ = 4.1 × 10^−8^, in agreement with the reported inefficiency of this phase of metastasis formation. For all analyzed cancers, the estimated bottleneck distribution has a large variance, indicating considerable variability of bottleneck severity within and between patients. To assess whether this flexibility of the model is necessary to explain the data, we repeated the entire data analysis, estimating a reduced model with single fixed bottleneck parameter rather than a bottleneck distribution (see S1 Text). The goodness of fit of the reduced model to the median survival data is comparable to the fit of the full model (S5 Fig) and the validation performance on the median survival data for the subset of patients with detected metastases (S7 Fig) is also similar. In contrast, however, both the goodness of fit to the metastasis detection data and the validation performance on the cancer death frequency data are worse for the reduced model (illustrated in S4 and S6 Figs), confirming the key importance of accounting for bottleneck variability.

### Predicted impact of treatment change on patient outcome

**Fig 5:**
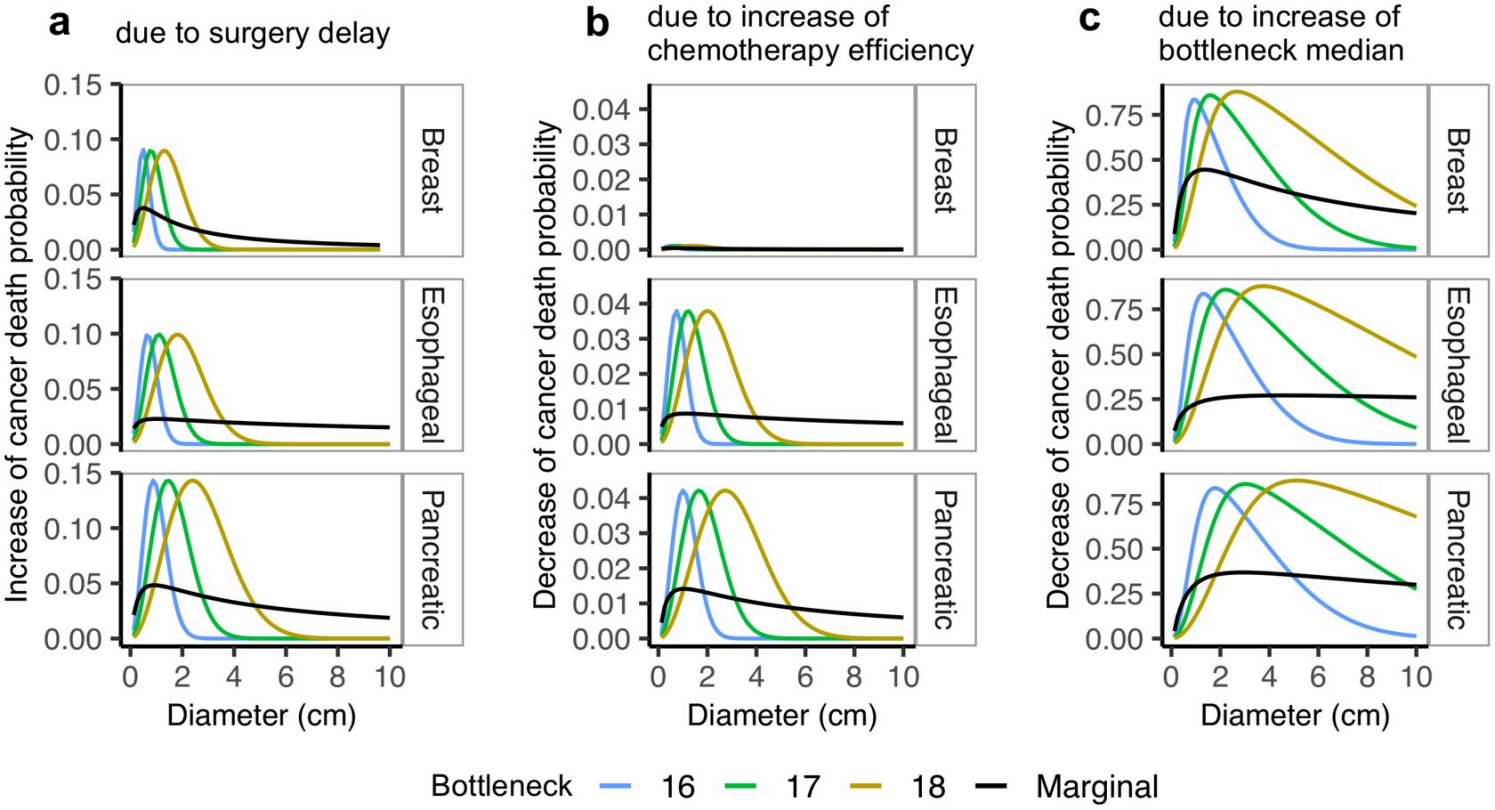
Predicted impact of treatment decisions. Change in cancer death probability (*y*-axis) due to treatment change depends on tumor diameter (*x*-axis) and bottleneck severity (colors). Black curves present the change in cancer death probability, marginalized over the bottleneck severity. Shown for the most impacted cancers: pancreatic (with the highest marginal impact of surgery delay and chemotherapy boost), breast (with the highest marginal impact of increasing bottleneck severity) and esophageal cancer (with the highest observed impact overall) (rows). The increase of cancer death probability due to surgery delay by 16 weeks (**a**), as well as decrease of cancer death probability due to increase of chemotherapy efficacy by 20% (**b**) are both much smaller than the decrease due to strengthening of the metastatic bottleneck by 20% (**c**). Surgery delay is modeled by allowing the tumor seed the metastases longer, chemotherapy efficiency boost is modeled by allowing chemotherapy removing more and larger metastases, while the strengthening the bottleneck is modeled by increasing the median bottleneck severity parameter.

Finally, we apply our model to predict impact of treatment decisions on patient survival (Fig 5 and S8 Fig). To this end, we consider how they will impact the probability of cancer-related death, both for patients with fixed exemplary bottleneck severity values, and for the entire population, where the impact is marginalized with respect to the bottleneck distribution. First, to evaluate the impact of possible surgery delay, we modified the model by effectively allowing the tumor to grow and seed the metastases *δ* = 16 weeks longer than the actual time of diagnosis (Fig 5a; using Eq 12 for fixed bottleneck severity and Eq 13 for marginalized bottleneck). Second, we evaluate the impact of increasing the efficiency of a systemic therapy, such as chemotherapy. This was modeled by allowing chemotherapy removing more and larger metastases, or equivalently increasing the expected age *a* at which a metastasis becomes irremovable by a factor *c*_1_ = 1.2 (i.e., by 20%) (Fig 5b; Eqs 14 and 15). Third, we evaluate a treatment strategy resulting in *c*_2_ = 1.2 (20%) increase of the median bottleneck severity (Fig 5c; Eqs 16 and 17). S8 Fig compares the results of the impact predictions for *δ* = 16 weeks with *δ* = 8 weeks, as well as for *c*_1_ = 1.2 with *c*_1_ = 1.1, and for *c*_2_ = 1.2 with *c*_2_ = 1.1. In all predictive equations, all other parameters were fixed to those estimated for the respective cancer types from the SEER data.

The model predicts that the impact of all three treatment decisions is tumor size-dependent, with lower impact for small and large tumors and larger impact for mid-sized tumors. A similar behavior, but only for the impact of surgery delay, was reported in mice [31]. Intuitively, decisions such as delay of surgery or modification of treatment efficacy may not matter for small tumors, which did not yet succeed to develop metastases, nor for large tumors, which already have acquired them. Importantly, different cancer types differ in the primary tumor sizes that are most sensitive for treatment decisions.

Besides the primary tumor size, our model suggests that the impact of treatment strategies depends also on the metastatic bottleneck, such that the peak of the impact is different for different bottleneck severities. For example, for fixed bottleneck severities (color curves in Fig 5a and S8 Fig), the largest predicted increase of cancer death probability due to surgery delay of 16 weeks is for head and neck cancer (S8 Fig): for a bottleneck severity of 16 cells and a tumor diameter of 0.625 cm, the increase is circa 0.19 and decays to 0 for larger tumors. For the same cancer type, but bottleneck severity 17, the peak impact of surgery delay is at a tumor diameter of 1.125 cm. The marginal (w.r.t. the bottleneck distribution) increase of cancer death probability due to surgery delay of 16 weeks (black curves in Fig 5a) does not exceed 0.05, and is the largest for pancreatic cancer and a tumor diameter of 0.875 cm. The estimated bottleneck distributions (Fig 4d) imply that the impact will vary both between and within patients. Since the individual bottleneck severities are unknown, our estimates of the marginal impact can be used to indicate how treatment decisions will affect most of the individuals.

We predict that a systemic therapy that would decrease the chances of successful metastatic colony initiation and tighten the median bottleneck by 20% (Fig 5c), would roughly have a 100-fold higher impact on patient survival than a 20% increase of chemotherapy efficacy (Fig 5b). Across all cancers and diameters, the largest obtainable marginal decrease in cancer death probability due to increase in chemotherapy efficiency by 20% is 0.01 (for pancreatic cancer and tumor diameter 1 cm). For a median bottleneck severity increase by 20%, the maximum marginal decrease of cancer death probability is 0.44, for breast cancer. Caution should be taken, however, when directly comparing the effects of tightening the metastatic bottleneck to the effect of boosting the chemotherapy. This is because the corresponding variables—the expected age at which a metastasis becomes irremovable and the the median bottleneck severity—have different meanings and live on different scales.

## Discussion

In this work, we provide a mathematical model for metastasis initiation, proposing the Allee effect as an explanation for the metastatic bottleneck. Furthermore, we estimate the severity of the bottleneck from epidemiological data for different cancers. The presented work extends current knowledge about metastasis formation in several ways. It is well known that tumor size influences prognosis, and tumor diameter is an important factor in the TNM staging system. The proposed model links tumor size to metastatic probability and provides mathematical expressions that describe the non-linear dependence of patient outcome on tumor size. We found that the size of the tumor is also predictive of how much a patient will benefit from a given treatment. Our model predictions indicate that only patients with not too large or too small tumors would benefit from additional treatments. Therefore, our model might be useful to support clinical decision making. In addition, we emphasize the importance to account for variability of metastasis initiation within and between patients. The model suggests that, apart from tumor size, also the individual bottleneck severity has a significant impact on patient response. The model predicts a high potential of treatment aiming at narrowing the metastatic bottleneck. Such bottleneck treatment would increase the chances of patient survival by reducing the chances to form a metastasis. Recent advances in immunological cancer treatment led to clinical trials of anticancer vaccines [43]. A bottleneck shift may be achieved in the future by such vaccines that strengthen the immune system against forming metastatic colonies, by reducing the number of circulating tumor cell clusters, or by such drugs that reduce cell adhesion in a cancer cell-specific manner.

We deliberately kept the proposed mathematical model as simple as possible, minimizing the number of free parameters. Except from modeling the distribution of the bottleneck severity, which is the most sensitive parameter in the model, other, less sensitive parameters are fixed to values derived from the literature or estimated as robust summary statistics of time to events. One example of a fixed parameter is the extravasation probability, which was set to 0.8 based on experiments in mice. Perturbations of this parameter change the results only sightly (S9 Fig). An example of a robust free parameter in the model is an average time to death from the acquisition of metastases. The model does not account, or only indirectly accounts for several phenomena that were proposed by previous studies. First, the model assumes only seeding of metastases with single, and not larger clusters of cells. Considering the possibility of seeding with clusters would result in many additional free parameters in the model, accounting for per-tumor cell, per-year release rates and extravasation probabilities for the clusters. Future work is required to address this issue, including experimental specification of parameters and extension of the model. According to the model, seeding with a cluster of several cells increases the probability of survival of the seeded metastasis (Fig 1c). Accordingly, we expect that modeling the initiation of metastases with cell clusters would result in an increased estimate of the median bottleneck severity. In this way, the model would compensate for the increase of the survival probability due to larger seeds by making the critical point harder to reach. The effect of metastatic latency is incorporated into the parameters of our model that correspond to the mean time of a metastasis to become detectable or lethal. Finally, our model is less expressive than several others, describing for example metastasis size distribution [44, 45] or metastatic growth. As such, it can be fit to and is predictive of epidemiological data, while more expressive models require model-tailored meticulous measurements of tumor burden. Despite its simplicity, our model is able to capture and be predictive of a surprising variety of aspects of clinical outcome. In summary, the presented model is a step forward in the understanding of metastasis formation, its bottleneck, and its impact on patient outcome.

## Supporting information

S1 Fig Epidemiological data from the SEER database [41] for ovarian cancer compared to an average of other analyzed cancer types (red). **a** For the other cancers, on average, the probability of metastasis detection (left) and the probability of cancer death (right) increase with tumor size at diagnosis. In contrast, ovarian cancer data do not follow these monotonic trends. **b** For the other cancers, on average, the median time to death for all patients (left) decreases with tumor size at diagnosis, but not for ovarian cancer. For the subset of patients who had metastases detected at diagnosis (right), the median time to death is generally much shorter and is not tumor-size dependent.
(PDF)

S2 Fig Fit and validation of the model on the SEER data, for ten additional cancer types (rows, ordered by the decreasing goodness of fit, or increasing RMSE), which are not featured in Fig 3 in the main text. Black points represent data records, blue lines show fitted, and red lines predicted curves. The model fits the data perfectly. **a** Metastasis detection probability (left panel) is compared to cancer death probability (right). For cancer death probability, up and down-oriented triangles present the upper and lower estimates of that variable from the data, respectively, while the black dots represent a Kaplan-Meier (KM) derived estimate. The predictions stay within the range of the estimators. **b** The median time to death data for all patients, (left panel) was used for model fitting and is tumor-size dependent. For a validation cohort of patients with metastases detected at diagnosis, the model correctly predicts a much shorter median time to death and that this time is almost constant across tumor diameters (right).
(PDF)

S3 Fig Summary of the model fit. For thirteen different cancer types, their fit to the data is measured using root mean squared error (RMSE). The cancer types (rows) are ordered by the increasing mean RMSE of the fit to quantile time to death data. **a** Low RMSE values indicate very good agreement of the model with metastasis detection probability (as visualized also in Fig 3a in the main text and S2a Fig, blue lines). The largest RMSE is obtained for pancreatic cancer, for which also the largest absolute metastasis detection probability is recorded (see S2a Fig). **b** Similarly good fit is obtained for quantile time to death data, for eleven different quantiles (x-axis). Larger RMSE values than for metastasis detection probability in (**a**) are due to the fact that quantile time to death is in measured in years (usually, several) and not probability values. The fit to 0.5-th quantile (median) is visualized also in Fig 3b in the main text and S2b Fig. The RMSE for other quantiles is comparable to the RMSE obtained for the median (0.50-th quantile) time to death data.
(PDF)

S4 Fig Fit of the reduced model on the metastasis detection data from the SEER database for thirteen cancer types. The reduced model is the same as the proposed model but with single fixed bottleneck severity parameter instead of distribution. Black points represent data records, blue lines show the fitted curves. In contrast to the excellent fit obtained by the proposed model (compare to Fig 3a left in the main text and S2a Fig left), the reduced model obtains a worse fit, especially for colon and colon mucinous cancer types.
(PDF)

S5 Fig Fit of the reduced model on the median survival data from the SEER database for thirteen cancer types. The reduced model is the same as the proposed model but with single fixed bottleneck severity parameter instead of distribution. Black points represent data records, blue lines show the fitted curves. For this data, the reduced model obtained a comparable fit as the proposed model (compare to Fig 3b left in the main text and S2b Fig left).
(PDF)

S6 Fig Poor validation of the reduced model on the cancer death frequency data from the SEER database, for thirteen cancer types. The reduced model is the same as the proposed model but with single fixed bottleneck severity parameter instead of distribution. Black points represent cancer death probability derived from the data records: up and down-oriented triangles present the upper and lower estimates of that variable from the data, respectively, while the black dots represent a Kaplan-Meier (KM) derived estimate. Red lines show predicted curves. The reduced model performs poorly in predicting the true values observed in the data, obtaining worse validation performance than the proposed model (compare to Fig 3a right in the main text and S2a Fig right).
(PDF)

S7 Fig Validation of the reduced model on the median survival data from the SEER database, for the subset of patients with detected metastases, and eight cancer types. For the remaining cancers the sample size was too small to compute the medians for more than one tumor diameter. The reduced model is the same as the proposed model but with single fixed bottleneck severity parameter instead of distribution. Black points represent median time to death for patients with metastases detected at diagnosis, derived from the data records. Red lines show predicted curves. For this data, compared to the proposed model (see Fig 3b right in the main text and S2b Fig right), the reduced model obtained a comparable validation performance.
(PDF)

S8 Fig Predicted impact of treatment decisions, for ten additional cancer types (rows, alphabetically ordered), which are not featured in Fig 4 in the main text. Change in cancer death probability (*y*-axis) due to treatment change depends on tumor diameter (*x*-axis) and bottleneck severity (colors). Black curves present the change in cancer death probability marginalized over the bottleneck severity. The increase of cancer death probability due to surgery delay by either 16 or 8 weeks (solid or dashed lines in **a**, respectively), as well as decrease of cancer death probability due to increase of chemotherapy efficacy by 20% (solid lines in **b**) or by 10% (dashed lines in **b**), are both much smaller than the decrease due to strengthening of the metastatic bottleneck by 20% (solid lines in **c**) or by 10% (dashed lines in **c**).
(PDF)

S9 Fig Results as presented in Fig 3 in the main text but for a model with an altered extravasation probability. **a, c, e** Metastasis detection probability (left panel) and cancer death probability (right). **b, d, f** The median time to death data for all patients, (left panel) and patients with metastases detected at diagnosis (right). **a, b** For extravasation probability 0.08. **c, d** For extravasation probability 0.4. **e, f** For extravasation probability 1. Compared to the model with extravasation probability 0.8, presented in Fig 3, the curves for the model with extravasation probability 0.08 (10 times smaller) are not as close to the data, but for extravasation probability 0.4 (two times smaller) or 1 (1.25 times larger) they are almost as close.
(PDF)

S1 Text Supplementary text. Comparison to a simpler model by Cisneros and Newman. Full derivation and extensions of the mathematical model of the metastasis bottleneck and patient outcome, with theoretical model generalizations. Processing epidemiological data. Model fitting and validation. Formulation of the reduced model.
(PDF)

S1 Table Per-cancer tumor growth rates fixed for thirteen modeled cancer types. For each cancer type, we list the tumor doubling time in days, denoted *D*, as found reported (usually as median or mean value across a measured population) in the cited references. The per year exponential growth rate *r* in our model, was then computed from those doubling times as ln(2) ⋅ 365.24/*D*. For several cancer types, we were unable to find measured doubling times. For those cancer types, we fixed the corresponding rates to ones measured in the most similar cancer type. Specifically, for endometrial cancer, we fixed the doubling rate to the rate reported in another female cancer, breast. For esophageal, gastric and bladder cancer types, we fixed the rate as reported for colon and rectum.
(PDF)

S2 Table Overview of analyzed cancer types with minimum follow up times and number of cases included after filtering from the SEER [41] database. For each cancer type, we provide a short name that is used throughout the manuscript. For each type, we defined a designated minimum follow-up time that ensures a sufficient number of patients for calculating representative statistics of frequency of metastasis detection, frequency of cancer death and quantile times to death. Generally, the minimum follow up times were a compromise between the requirement of long monitoring time after diagnosis, and the resulting sample sizes for each cancer. The minimum follow-up time differs between different cancer types, reflecting their clinical behavior. For example, for more aggressive cancers, such as pancreatic and esophageal cancer, which have a poor prognosis, a shorter minimum follow-up time was allowed. In contrast, for breast cancer, where the ten year survival rate for all stages is nearly 80%, the minimum follow-up was set to 20 years. This still allowed for a very large sample size of 35949 patients, since breast cancer is one of the most common cancer types.
(PDF)

S3 Table Significance analysis of the monotonic dependence of clinical variables on tumor size. For each cancer type (rows), the p-values in a two-sided Mann-Kendall trend test are reported. Table entries in red contain p-values lower than 0.01. Light red entries contain p-values lower than 0.05. For metastasis detection probability, cancer death probability and median time to death, the monotonic trend is significant for all cancers, except ovarian and with exception of cancer death probability for esophageal and median time to death for colon mucinous cancer. The same results hold for false discovery rate (FDR) computed from p-values using Benjamini-Hochberg correction for 14 tests (first four columns) or without correction (last four columns). NA values in the “Median time to death for patients with mets” column indicate that, due to too small patient sample sizes, there is no such data available.
(PDF)

## References

[1] Sporn MB. The war on cancer. Lancet. 1996;347(9012):1377–1381. 10.1016/S0140-6736(96)91015-6 8637346

[2] Hanahan D, Weinberg RA. Hallmarks of cancer: the next generation. Cell. 2011;144(5):646–674. 10.1016/j.cell.2011.02.013 21376230

[3] Fidler IJ. The pathogenesis of cancer metastasis: the ‘seed and soil’ hypothesis revisited. Nature Rev Cancer. 2003;3:453–458. 10.1038/nrc1098 12778135

[4] Chambers AF, Groom AC, MacDonald IC. Dissemination and growth of cancer cells in metastatic sites. Nature Rev Cancer. 2002;2:563–572. 10.1038/nrc865 12154349

[5] Nguyen DX, Bos PD, Massague J. Metastasis: from dissemination to organ-specific colonization. Nature Rev Cancer. 2009;9:274–284. 10.1038/nrc2622 19308067

[6] Chaffer CL, Weinberg RA. A perspective on cancer cell metastasis. Science. 2011;331:1559–1564. 10.1126/science.1203543 21436443

[7] Nguyen DX, Massague J. Genetic determinants of cancer metastasis. Nature Rev Genet. 2007;8:341–352. 10.1038/nrg2101 17440531

[8] Liu W. Copy number analysis indicates monoclonal origin of lethal metastatic prostate cancer. Nature Med. 2009;15:559–565. 10.1038/nm.1944 19363497 PMC2839160

[9] Navin N. Tumour evolution inferred by single-cell sequencing. Nature. 2011;472:90–94. 10.1038/nature09807 21399628 PMC4504184

[10] Hosseini H, Obradovi? MM, Hoffmann M, Harper KL, Sosa MS, Werner-Klein M, et al Early dissemination seeds metastasis in breast cancer. Nature. 2016 10.1038/nature20785 PMC5390864 27974799

[11] Harper KL, Sosa MS, Entenberg D, Hosseini H, Cheung JF, Nobre R, et al Mechanism of early dissemination and metastasis in Her2(+) mammary cancer. Nature. 2016 10.1038/nature20609 PMC5471138 27974798

[12] Luzzi KJ. Multistep nature of metastatic inefficiency: dormancy of solitary cells after successful extravasation and limited survival of early micrometastases. Am J Pathol. 1998;153:865–873. 10.1016/S0002-9440(10)65628-3 9736035 PMC1853000

[13] Cameron MD. Temporal progression of metastasis in lung: cell survival, dormancy, and location dependence of metastatic inefficiency. Cancer Res. 2000;60:2541–2546. 10811137

[14] Varghese HJ. Activated Ras regulates the proliferation/apoptosis balance and early survival of developing micrometastases. Cancer Res. 2002;62:887–891. 11830548

[15] Fidler I. Metastasis: guantitative analysis of distribution and fate of tumor emboli labeled with 125 I-5-iodo-2’-deoxyuridine. Journal of the National Cancer Institute. 1970;45:773–82. 5513503

[16] Weiss L. Metastatic inefficiency. Adv Cancer Res. 1990;54:159–211. 10.1016/S0065-230X(08)60811-8 1688681

[17] Wong CW, Lee A, Shientag L, Yu J, Dong Y, Kao G, et al Apoptosis: An Early Event in Metastatic Inefficiency. Cancer Research. 2001;61(1):333–338. 11196183

[18] Paget S. The distribution of secondary growths in cancer of the breast. 1889 Cancer Metastasis Rev. 1989;8:98–101. 2673568

[19] Langley RR, Fidler IJ. The seed and soil hypothesis revisited–the role of tumor-stroma interactions in metastasis to different organs. Int J Cancer. 2011;128(11):2527–2535. 10.1002/ijc.26031 21365651 PMC3075088

[20] Liotta LA, Saidel MG, Kleinerman J. The significance of hematogenous tumor cell clumps in the metastatic process. Cancer Res. 1976;36(3):889–894. 1253177

[21] Aceto N, Bardia A, Miyamoto DT, Donaldson MC, Wittner BS, Spencer JA, et al Circulating tumor cell clusters are oligoclonal precursors of breast cancer metastasis. Cell. 2014;158(5):1110–1122. 10.1016/j.cell.2014.07.013 25171411 PMC4149753

[22] Liotta LA, Kleinerman J, Saidel GM. Quantitative relationships of intravascular tumor cells, tumor vessels, and pulmonary metastases following tumor implantation. Cancer Res. 1974;34(5):997–1004. 4841969

[23] Saidel GM, Liotta LA, Kleinerman J. System dynamics of metastatic process from an implanted tumor. J Theor Biol. 1976;56(2):417–434. 10.1016/S0022-5193(76)80083-5 1271828

[24] Liotta LA, Saidel GM, Kleinerman J. Stochastic model of metastases formation. Biometrics. 1976;32(3):535–550. 10.2307/2529743 963169

[25] Hartung N, Mollard S, Barbolosi D, Benabdallah A, Chapuisat G, Henry G, et al Mathematical modeling of tumor growth and metastatic spreading: validation in tumor-bearing mice. Cancer Res. 2014;74(22):6397–6407. 10.1158/0008-5472.CAN-14-0721 25217520

[26] Avanzini S, Antal T. Cancer recurrence times from a branching process model. PLOS Computational Biology. 2019;15(11):e1007423 10.1371/journal.pcbi.1007423 31751332 PMC6871767

[27] Michor F, Nowak MA, Iwasa Y. Stochastic dynamics of metastasis formation. J Theor Biol. 2006;240(4):521–530. 10.1016/j.jtbi.2005.10.021 16343545

[28] Dingli D, Michor F, Antal T, Pacheco JM. The emergence of tumor metastases. Cancer Biol Ther. 2007;6(3):383–390. 10.4161/cbt.6.3.3720 17312385

[29] Haeno H, Michor F. The evolution of tumor metastases during clonal expansion. J Theor Biol. 2010;263(1):30–44. 10.1016/j.jtbi.2009.11.005 19917298 PMC3713519

[30] Haeno H, Gonen M, Davis MB, Herman JM, Iacobuzio-Donahue CA, Michor F. Computational Modeling of Pancreatic Cancer Reveals Kinetics of Metastasis Suggesting Optimum Treatment Strategies. Cell. 2012;148(1-2):362–375. 10.1016/j.cell.2011.11.060. 22265421 PMC3289413

[31] Benzekry S, Tracz A, Mastri M, Corbelli R, Barbolosi D, Ebos JM. Modeling Spontaneous Metastasis following Surgery: An In Vivo-In Silico Approach. Cancer Res. 2016;76(3):535–547. 10.1158/0008-5472.CAN-15-1389 26511632 PMC5846333

[32] Cisneros LH, Newman TJ. Quantifying metastatic inefficiency: rare genotypes versus rare dynamics. Phys Biol. 2014;11(4):046003 10.1088/1478-3975/11/4/046003 25033031

[33] Angela Moles MW. Seedling survival and seed size: A synthesis of the literature. Journal of Ecology. 2004;92:372383.

[34] Resh Vincent H C RT, editor. Encyclopedia of Insects. Academic Press; 2009.

[35] Courchamp F, Berec L. Allee effects in ecology and conservation Oxford biology. Oxford: Oxford University Press; 2008.

[36] Johnson KE, Howard G, Mo W, Strasser MK, Lima EABF, Huang S, et al Cancer cell population growth kinetics at low densities deviate from the exponential growth model and suggest an Allee effect. PLoS Biol. 2019;17(8):e3000399 10.1371/journal.pbio.3000399 31381560 PMC6695196

[37] Brierley JD, Gospodarowicz MK, Wittekind Ce, editors. TNM Classification of malignant tumours. 8th ed Chichester, West Sussex, UK; Hoboken, NJ: John Wiley & Sons, Inc; 2017.

[38] Koscielny S. Breast cancer: relationship between the size of the primary tumour and the probability of metastatic dissemination. Br J Cancer. 1984;49:709–715. 10.1038/bjc.1984.112 6733019 PMC1976833

[39] Engel J, Eckel R, Kerr J, Schmidt M, Furstenberger G, Richter R, et al The process of metastasisation for breast cancer. Eur J Cancer. 2003;39(12):1794–1806. 10.1016/S0959-8049(03)00422-2 12888376

[40] DeVita VT, Young RC, Canellos GP. Combination versus single agent chemotherapy: a review of the basis for selection of drug treatment of cancer. Cancer. 1975;35(1):98–110. 10.1002/1097-0142(197501)35:1<98::AID-CNCR2820350115>3.0.CO;2-B 162854

[41] SEER. Surveillance, Epidemiology, and End Results Program (www.seer.cancer.gov) Research Data (1973-2013), National Cancer Institute, DCCPS, Surveillance Research Program, Surveillance Systems Branch; released in Nov 2015.

[42] Lengyel E. Ovarian cancer development and metastasis. Am J Pathol. 2010;177(3):1053–1064. 10.2353/ajpath.2010.100105 20651229 PMC2928939

[43] Melero I, Gaudernack G, Gerritsen W, Huber C, Parmiani G, Scholl S, et al Therapeutic vaccines for cancer: an overview of clinical trials. Nat Rev Clin Oncol. 2014;11(9):509–524. 10.1038/nrclinonc.2014.111 25001465

[44] Hanin L, Rose J, Zaider M. A stochastic model for the sizes of detectable metastases. J Theor Biol. 2006;243(3):407–417. 10.1016/j.jtbi.2006.07.005 16930629

[45] Iwata K, Kawasaki K, Shigesada N. A dynamical model for the growth and size distribution of multiple metastatic tumors. J Theor Biol. 2000;203(2):177–186. 10.1006/jtbi.2000.1075 10704301

