# Supplementary figures and images for: A mathematical model of the metastatic bottleneck predicts patient outcome and response to cancer treatment
Source: PLoS Comput Biol. 2020 Oct 2;16(10):e1008056. doi: 10.1371/journal.pcbi.1008056 (PMC7591057; doi:10.1371/journal.pcbi.1008056)

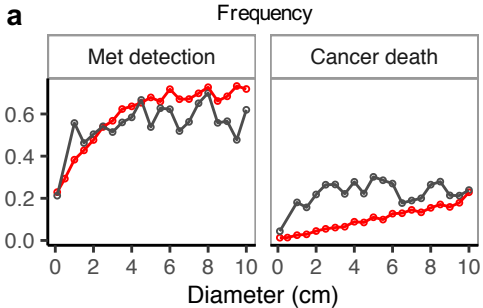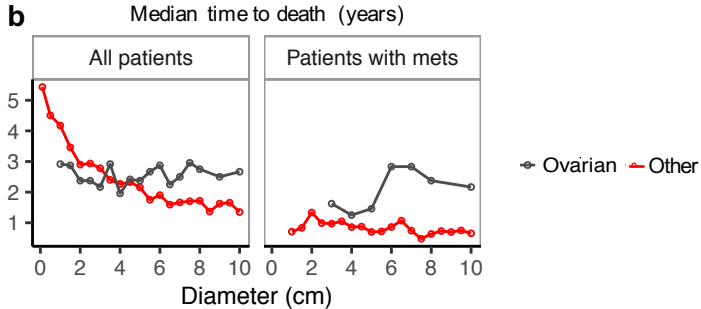

Supplement: S1 Fig — a For the other cancers, on average, the probability of metastasis detection (left) and the probability of cancer death (right) increase with tumor size at diagnosis. In contrast, ovarian cancer data do not follow these monotonic trends. b For the other cancers, on average, the median time to death for all patients (left) decreases with tumor size at diagnosis, but not for ovarian cancer. For the subset of patients who had metastases detected at diagnosis (right), the median time to death is generally much shorter and is not tumor-size dependent. (PDF) [file pcbi.1008056.s001.pdf]

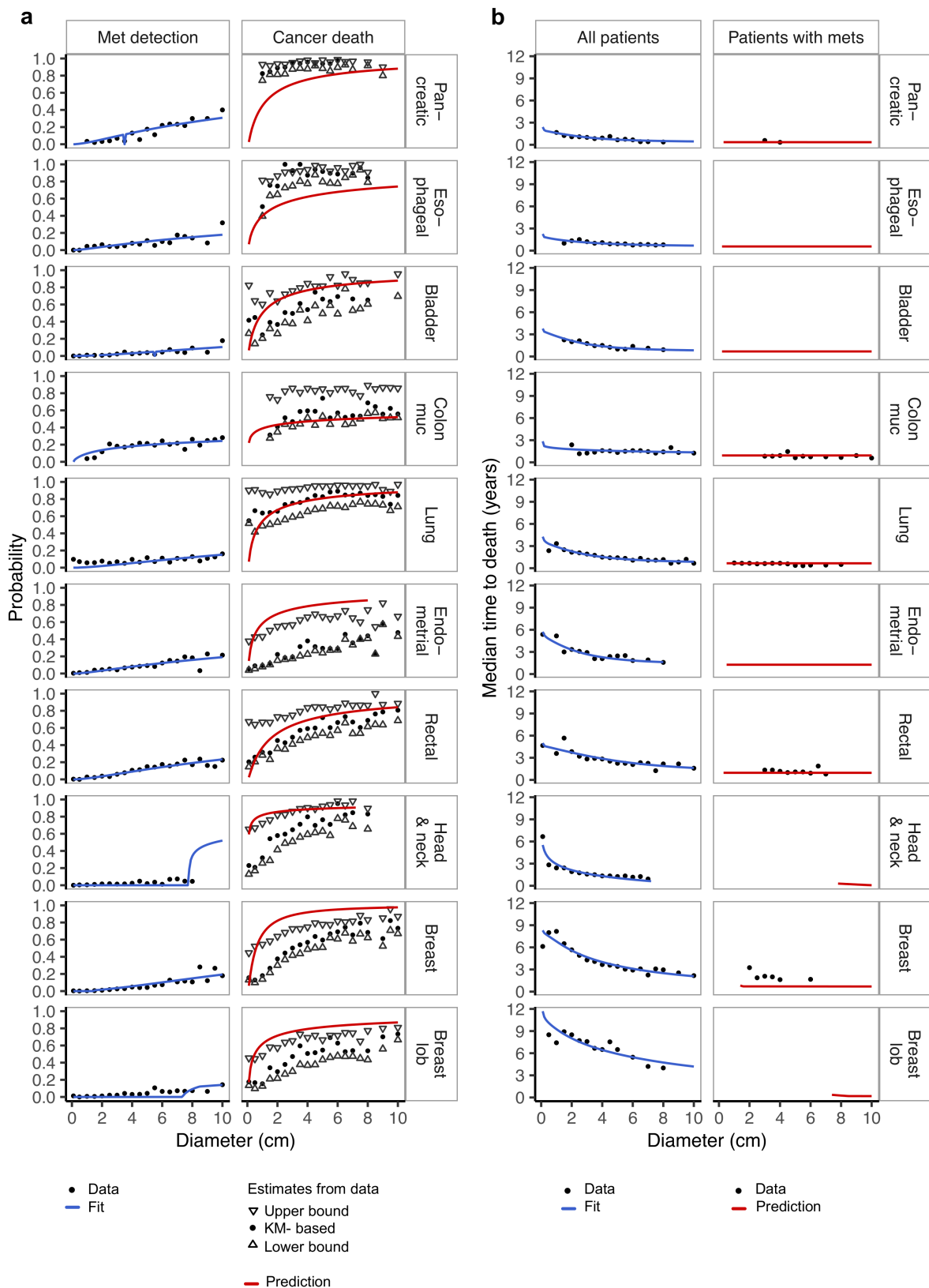

Supplement: S2 Fig — Black points represent data records, blue lines show fitted, and red lines predicted curves. The model fits the data perfectly. a Metastasis detection probability (left panel) is compared to cancer death probability (right). For cancer death probability, up and down-oriented triangles present the upper and lower estimates of that variable from the data, respectively, while the black dots represent a Kaplan-Meier (KM) derived estimate. The predictions stay within the range of the estimators. b The median time to death data for all patients, (left panel) was used for model fitting and is tumor-size dependent. For a validation cohort of patients with metastases detected at diagnosis, the model correctly predicts a much shorter median time to death and that this time is almost constant across tumor diameters (right). (PDF) [file pcbi.1008056.s002.pdf]

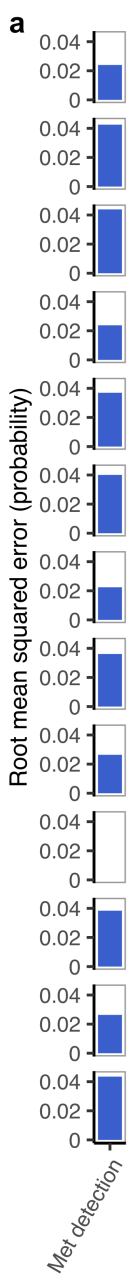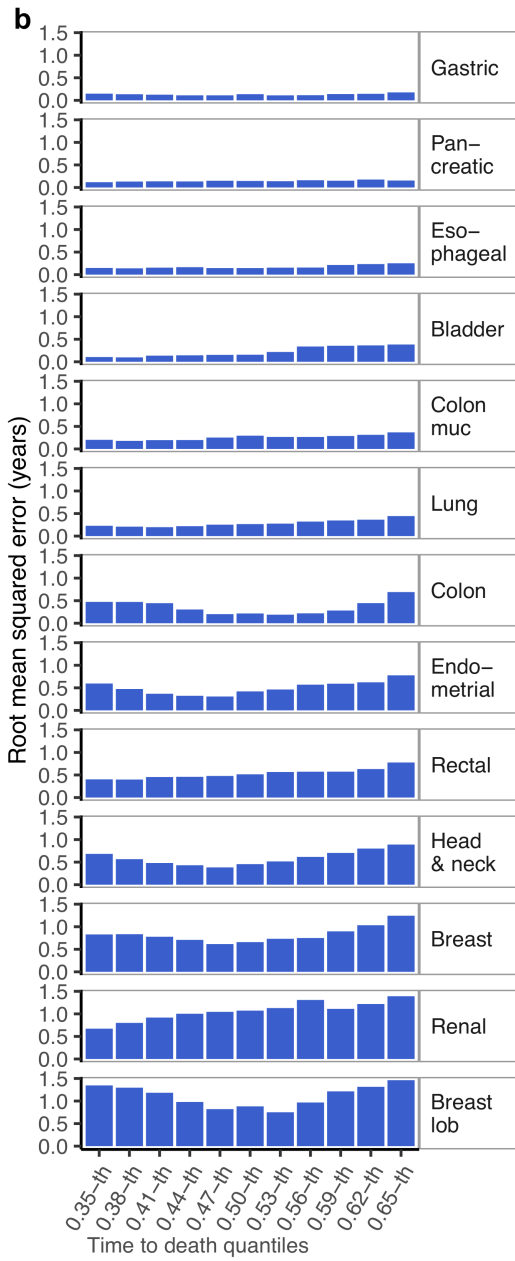

Supplement: S3 Fig — For thirteen different cancer types, their fit to the data is measured using root mean squared error (RMSE). The cancer types (rows) are ordered by the increasing mean RMSE of the fit to quantile time to death data. a Low RMSE values indicate very good agreement of the model with metastasis detection probability (as visualized also in Fig 3a in the main text and S2a Fig, blue lines). The largest RMSE is obtained for pancreatic cancer, for which also the largest absolute metastasis detection probability is recorded (see S2a Fig). b Similarly good fit is obtained for quantile time to death data, for eleven different quantiles (x-axis). Larger RMSE values than for metastasis detection probability in (a) are due to the fact that quantile time to death is in measured in years (usually, several) and not probability values. The fit to 0.5-th quantile (median) is visualized also in Fig 3b in the main text and S2b Fig. The RMSE for other quantiles is comparable to the RMSE obtained for the median (0.50-th quantile) time to death data. (PDF) [file pcbi.1008056.s003.pdf]

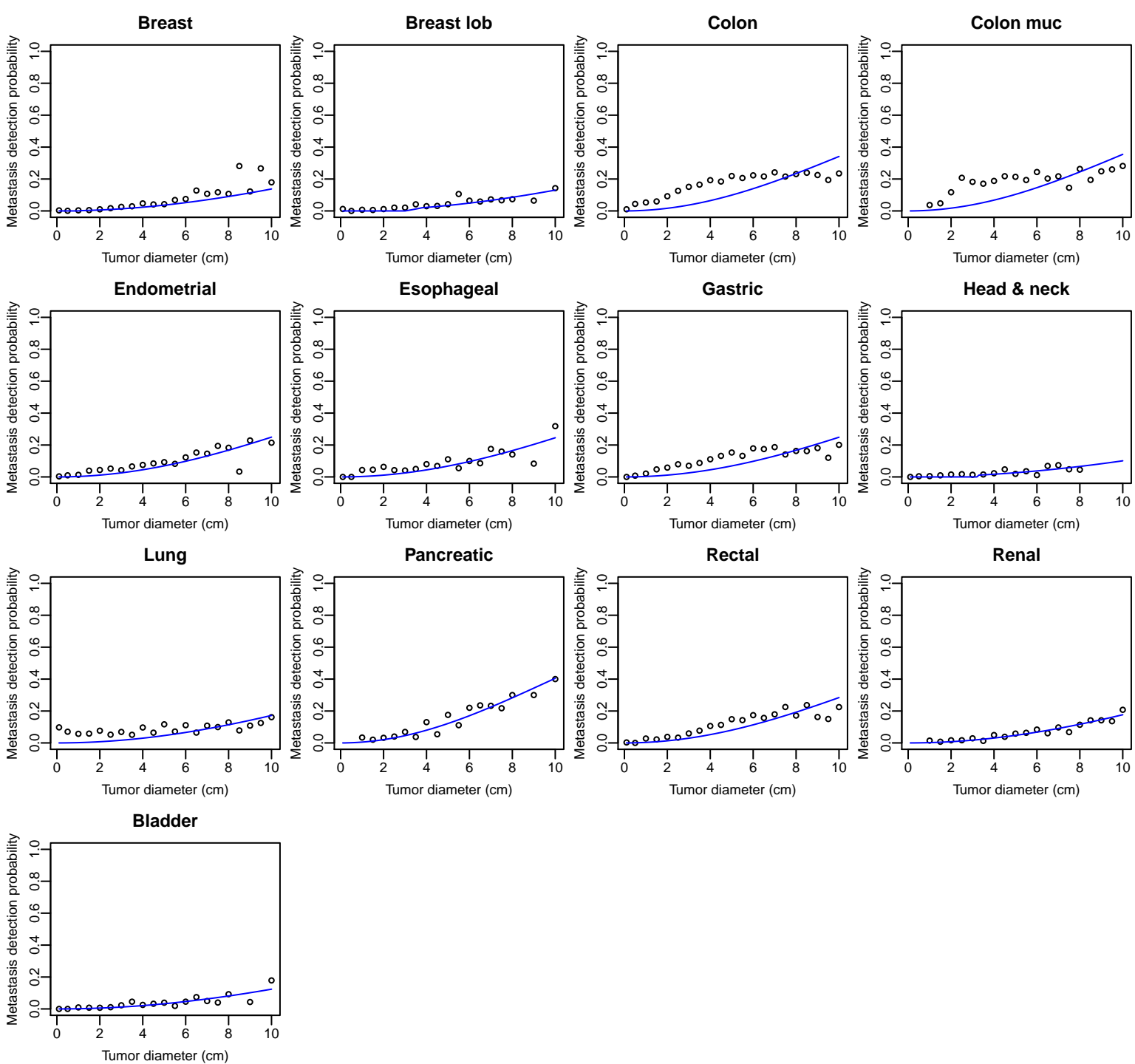

Supplement: S4 Fig — The reduced model is the same as the proposed model but with single fixed bottleneck severity parameter instead of distribution. Black points represent data records, blue lines show the fitted curves. In contrast to the excellent fit obtained by the proposed model (compare to Fig 3a left in the main text and S2a Fig left), the reduced model obtains a worse fit, especially for colon and colon mucinous cancer types. (PDF) [file pcbi.1008056.s004.pdf]

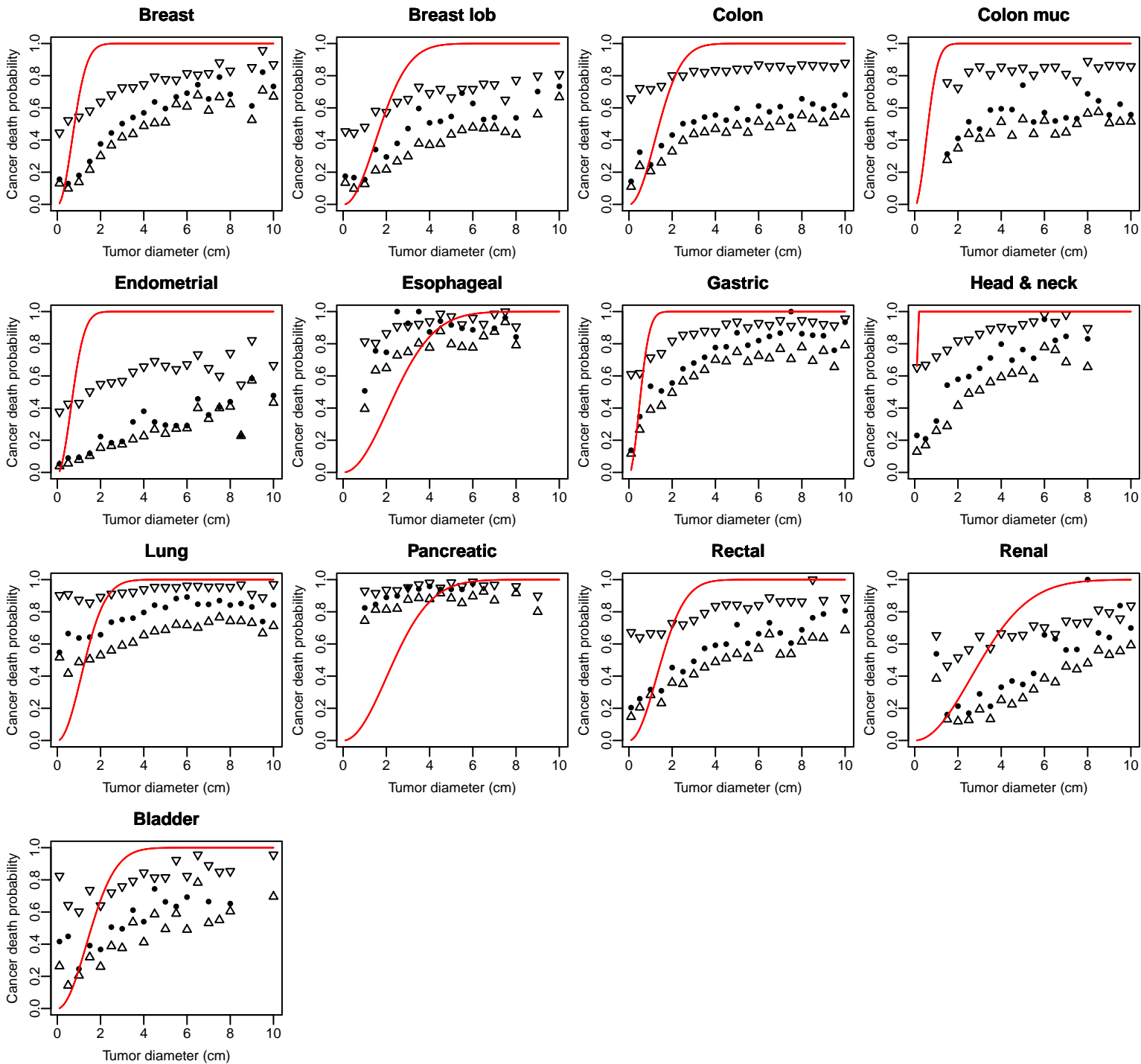

Supplement: S6 Fig — The reduced model is the same as the proposed model but with single fixed bottleneck severity parameter instead of distribution. Black points represent cancer death probability derived from the data records: up and down-oriented triangles present the upper and lower estimates of that variable from the data, respectively, while the black dots represent a Kaplan-Meier (KM) derived estimate. Red lines show predicted curves. The reduced model performs poorly in predicting the true values observed in the data, obtaining worse validation performance than the proposed model (compare to Fig 3a right in the main text and S2a Fig right). (PDF) [file pcbi.1008056.s006.pdf]

Breast

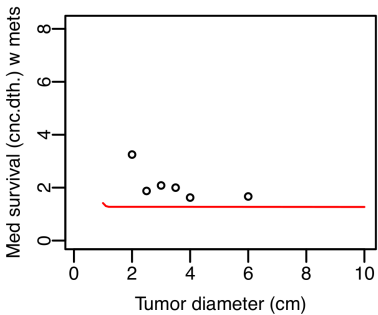

Colon

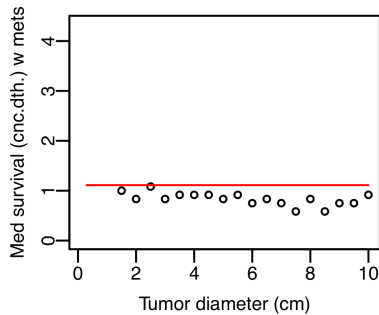

Colon muc

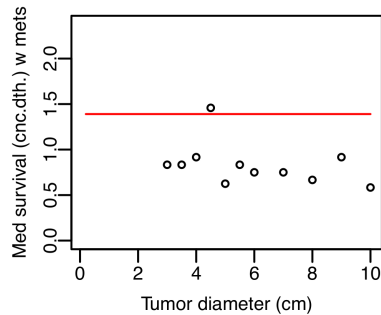

Gastric

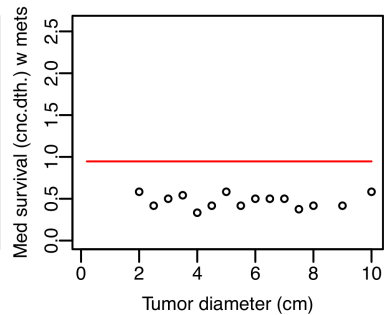

Lung

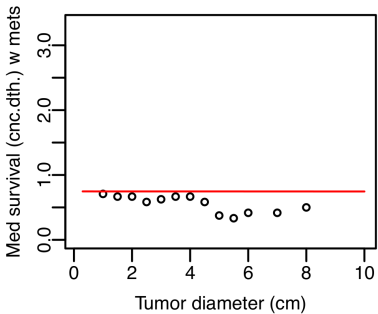

Pancreatic

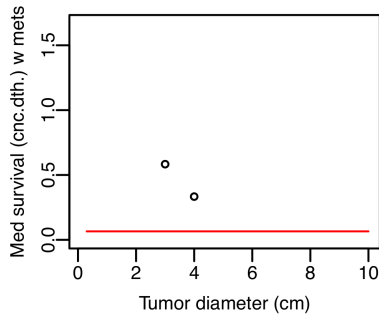

Rectal

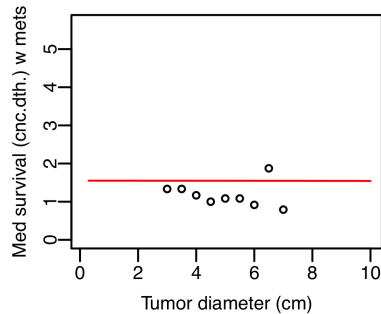

Renal

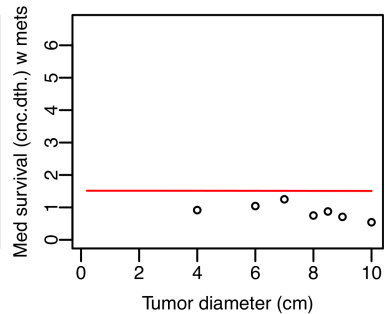

Supplement: S7 Fig — For the remaining cancers the sample size was too small to compute the medians for more than one tumor diameter. The reduced model is the same as the proposed model but with single fixed bottleneck severity parameter instead of distribution. Black points represent median time to death for patients with metastases detected at diagnosis, derived from the data records. Red lines show predicted curves. For this data, compared to the proposed model (see Fig 3b right in the main text and S2b Fig right), the reduced model obtained a comparable validation performance. (PDF) [file pcbi.1008056.s007.pdf]

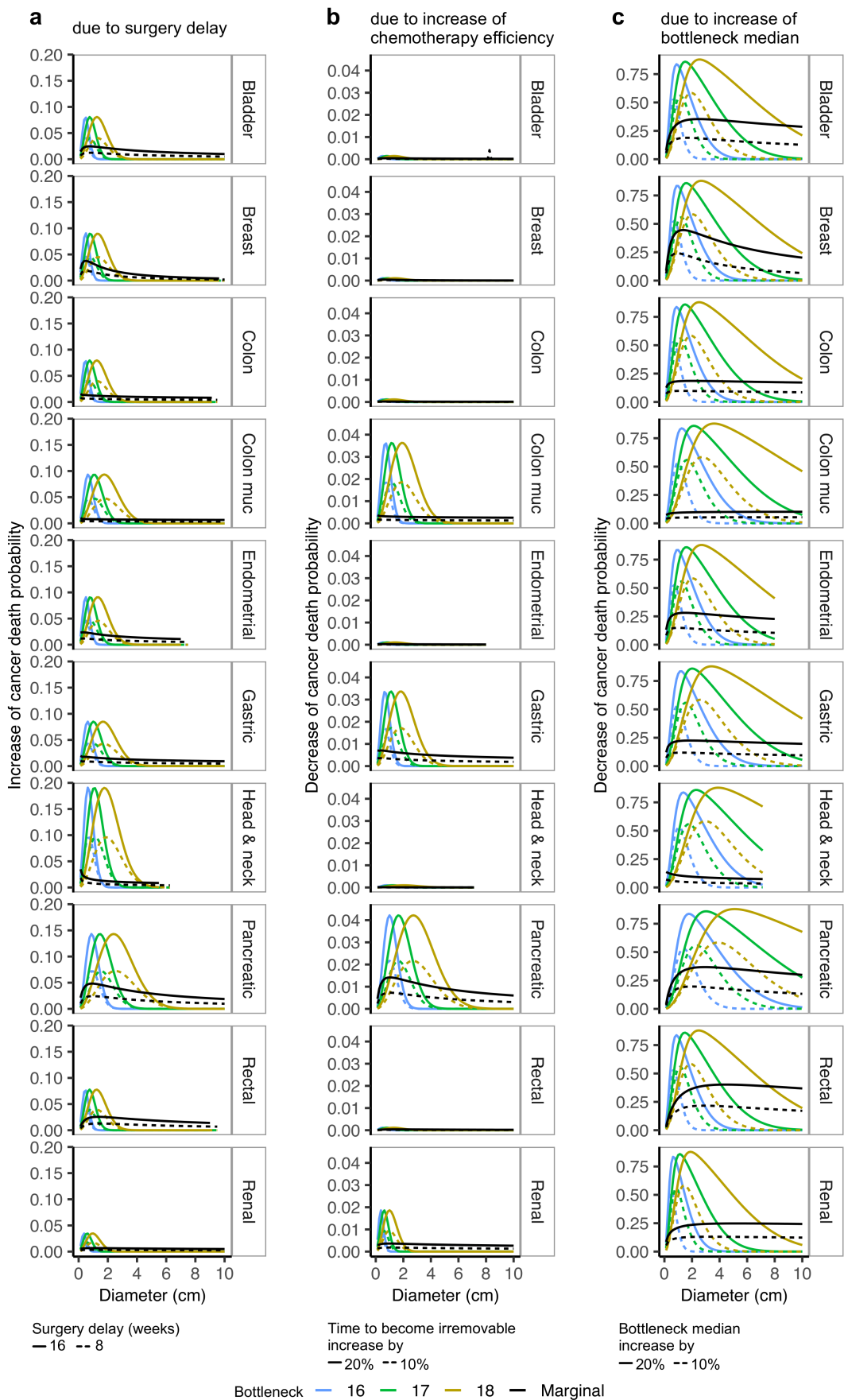

Supplement: S8 Fig — Change in cancer death probability (y-axis) due to treatment change depends on tumor diameter (x-axis) and bottleneck severity (colors). Black curves present the change in cancer death probability marginalized over the bottleneck severity. The increase of cancer death probability due to surgery delay by either 16 or 8 weeks (solid or dashed lines in a, respectively), as well as decrease of cancer death probability due to increase of chemotherapy efficacy by 20% (solid lines in b) or by 10% (dashed lines in b), are both much smaller than the decrease due to strengthening of the metastatic bottleneck by 20% (solid lines in c) or by 10% (dashed lines in c). (PDF) [file pcbi.1008056.s008.pdf]
