# Supplementary material for: A mathematical model of the metastatic bottleneck predicts patient outcome and response to cancer treatment
Source: PLoS Comput Biol. 2020 Oct 2;16(10):e1008056. doi: 10.1371/journal.pcbi.1008056 (PMC7591057; doi:10.1371/journal.pcbi.1008056)
Supplement: S5 Fig — The reduced model is the same as the proposed model but with single fixed bottleneck severity parameter instead of distribution. Black points represent data records, blue lines show the fitted curves. For this data, the reduced model obtained a comparable fit as the proposed model (compare to Fig 3b left in the main text and S2b Fig left). (PDF) [file pcbi.1008056.s005.pdf]

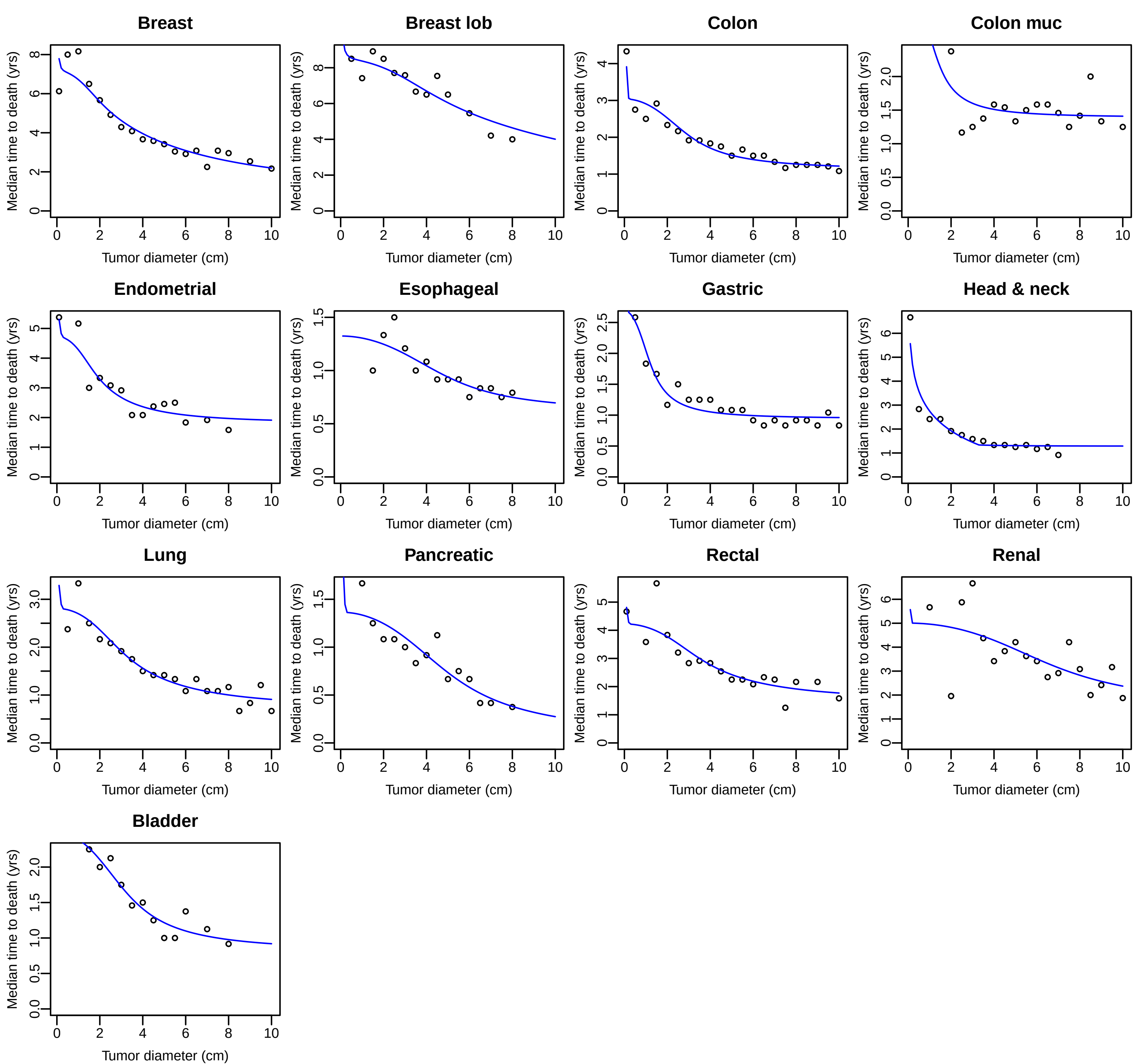
Breast
Median time to death (yrs)
Tumor diameter (cm)
Breast lob
Median time to death (yrs)
Tumor diameter (cm)
Colon
Median time to death (yrs)
Tumor diameter (cm)
Colon muc
Median time to death (yrs)
Tumor diameter (cm)
Endometrial
Median time to death (yrs)
Tumor diameter (cm)
Esophageal
Median time to death (yrs)
Tumor diameter (cm)
Gastric
Median time to death (yrs)
Tumor diameter (cm)
Head & neck
Median time to death (yrs)
Tumor diameter (cm)
Lung
Median time to death (yrs)
Tumor diameter (cm)
Pancreatic
Median time to death (yrs)
Tumor diameter (cm)
Rectal
Median time to death (yrs)
Tumor diameter (cm)
Renal
Median time to death (yrs)
Tumor diameter (cm)
Bladder
Median time to death (yrs)
Tumor diameter (cm)
