# Supplementary material for: A mathematical model of the metastatic bottleneck predicts patient outcome and response to cancer treatment
Source: PLoS Comput Biol. 2020 Oct 2;16(10):e1008056. doi: 10.1371/journal.pcbi.1008056 (PMC7591057; doi:10.1371/journal.pcbi.1008056)
Supplement: S9 Fig — a, c, e Metastasis detection probability (left panel) and cancer death probability (right). b, d, f The median time to death data for all patients, (left panel) and patients with metastases detected at diagnosis (right). a, b For extravasation probability 0.08. c, d For extravasation probability 0.4. e, f For extravasation probability 1. Compared to the model with extravasation probability 0.8, presented in Fig 3, the curves for the model with extravasation probability 0.08 (10 times smaller) are not as close to the data, but for extravasation probability 0.4 (two times smaller) or 1 (1.25 times larger) they are almost as close. (PDF) [file pcbi.1008056.s009.pdf]

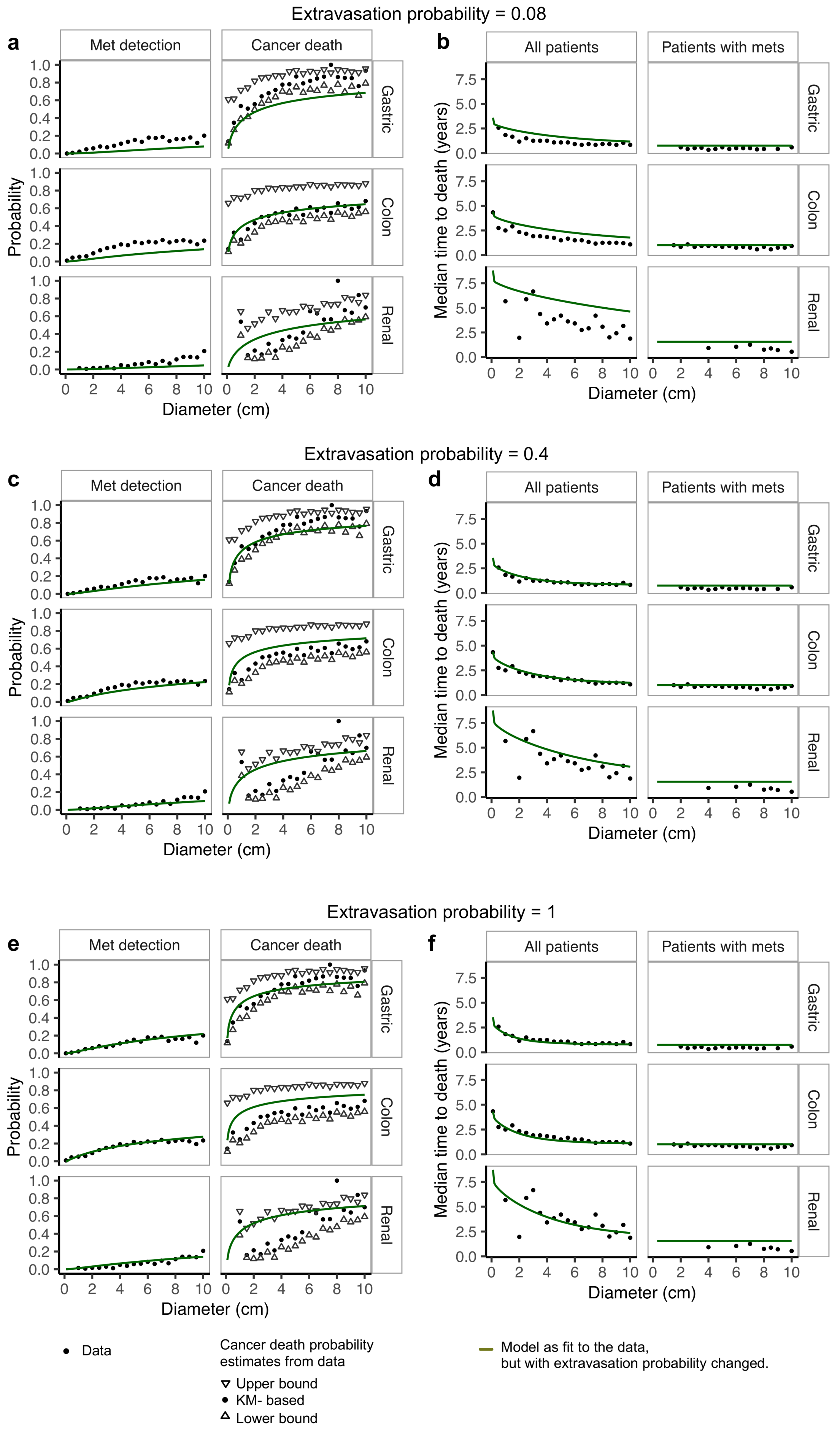

Extravasation probability = 0.08
a
Met detection
Cancer death
Gastric
Colon
Renal
Probability
Diameter (cm)
b
All patients
Patients with mets
Median time to death (years)
Diameter (cm)
Extravasation probability = 0.4
c
d
Extravasation probability = 1
e
f
Data
Cancer death probability estimates from data
Upper bound
KM- based
Lower bound
Model as fit to the data, but with extravasation probability changed.
