# Supplementary material for: A mathematical model of the metastatic bottleneck predicts patient outcome and response to cancer treatment
Source: PLoS Comput Biol. 2020 Oct 2;16(10):e1008056. doi: 10.1371/journal.pcbi.1008056 (PMC7591057; doi:10.1371/journal.pcbi.1008056)
Supplement: S1 Text — Comparison to a simpler model by Cisneros and Newman. Full derivation and extensions of the mathematical model of the metastasis bottleneck and patient outcome, with theoretical model generalizations. Processing epidemiological data. Model fitting and validation. Formulation of the reduced model. (PDF) [file pcbi.1008056.s010.pdf]

A MATHEMATICAL MODEL OF THE METASTATIC BOTTLENECK PREDICTS PATIENT OUTCOME AND RESPONSE TO CANCER TREATMENT

SUPPLEMENTARY TEXT

EWA SZCZUREK[1], TYLL KRÜGER[2], BARBARA KLINK[3,4], NIKO BEERENWINKEL[5,6,*]

1 Faculty of Mathematics, Informatics and Mechanics, University of Warsaw, Warsaw, Poland
2 Faculty of Electronics, Wrocław University of Science and Technology, Wrocław, Poland
3 Institute for Clinical Genetics, Faculty of Medicine Carl Gustav Carus, Technische Universität Dresden, Dresden, Germany
4 National Center of Genetics, Laboratoir national de santé, Dudelange, Luxembourg
5 Department of Biosystems Science and Engineering, ETH Zurich, Basel, Switzerland
6 SIB Swiss Institute of Bioinformatics, Basel, Switzerland

Contents

Comparison to a simpler model by Cisneros and Newman 2
Full derivation and extensions of the mathematical model of the metastasis bottleneck and patient outcome, with theoretical model generalizations 2
Derivation of the metastasis success probability 2
Consideration of a truncated birth to death rates ratio 4
Generalization of the metastasis initiation model 4
Full derivation of the dependence of metastasis probability on tumor size and bottleneck severity. 6
Full derivation of the post-surgical cancer death probability for patients diagnosed with given tumor size. 7
Full derivation of the quantile time to death from cancer for patients with given tumor size. 8
Processing epidemiological data 9
Model fitting and validation 10
Model fitting. 10
Validation on an independent dataset. 10
Formulation of the reduced model 10
References 11

Comparison to a simpler model by Cisneros and Newman

This work brings several improvements over a stochastic process model of metastatic colonization assuming a constant proliferation-to-death rate ratio, proposed previously by Cisneros and Newman [1].

First, according to the model of [1], until the colony reaches a certain size, the proliferation-to-death rate ratio is assumed to be a constant smaller than one, resulting in a sub-critical time-homogeneous process. For such a process, the probability of colony survival is zero. Therefore, upon reaching the given size, the process is switched abruptly to supercritical, by changing the proliferation-to-death ratio to another constant larger than one. Such a presumed sudden switch that occurs for all cells in the colony is very unlikely to occur in nature. By contrast, our model provides a more biologically plausible mechanism by gradually changing the birth-death ratio as a function of colony size. Consequently, according to our model, the probability of colony survival gradually increases from close to zero towards one with each additional cell in the colony (Fig. 1c in the main text).

Most importantly, the model of Cisneros and Newman [1] did not account for the variability of the critical colony size, or equivalently the metastatic bottleneck within and between patients, which is a key assumption of our model. Bottleneck variability is expected due to multiple biological reasons, such as differences in immune system strengths among patients and between different secondary sites, as well as genetic and phenotypic properties of the tumor seeding cells. In our model, the bottleneck severity, which determines the metastatic success probabilities via $s_1(b) = \exp(-b)$ and is thus an extremely sensitive parameter, is modeled with a probability distribution and not just by the mean value, as we do with other parameters. The importance of accounting for the bottleneck variability is demonstrated by a poor performance of a reduced model, formulated below, with a single fixed bottleneck parameter rather than a bottleneck distribution (Fig. S4-S7).

Finally, also in contrast to our model, Cisneros and Newman [1] did not account for the dependence of metastasis occurrence on the primary tumor growth and its impact on patient outcome, and was not fit to patient data.

Full derivation and extensions of the mathematical model of the metastasis bottleneck and patient outcome, with theoretical model generalizations

Derivation of the metastasis success probability. Consider the metastasis initiation model introduced in the main text, where for colony size i the proliferation rate is λ_i and the death rate is μ_i, and we assume

$$\frac{\lambda_i}{\mu_i} = \frac{i}{b}. \tag{1}$$

We will now show that for this model the metastasis success probability s_1, equal to the probability that the colony starting from single cell will not go extinct, is given by

$$s_1 = \exp(-b), \tag{2}$$

while the probability of colony survival starting from $i > 1$ cells equals

$$s_i = F_{Pois}(i-1; b),$$

where $F_{Pois}(i; b)$ denotes a cumulative Poisson distribution function with parameter b, evaluated at value i.

In a birth and death chain with a single absorbing state $i = 0$, the probability of success of the metastasis starting from state i can be computed with the recursion [2, 3]

$$s_i = (1 - p_i)\, s_{i-1} + p_i s_{i+1},$$

where p_i denotes the probability of jumping from the state of i to $i + 1$ cells

$$p_i = \frac{\lambda_i}{\lambda_i + \mu_i}.$$

Setting $a_i = \frac{1}{p_i} - 1$, with $s_0 = 0$, and rearranging terms we obtain

$$s_{i+1} - s_i = a_i\,(s_i - s_{i-1}) = \prod_{l=1}^{i} a_l s_1.$$

Hence,

$$s_{i+1} - s_i + s_i - s_{i-1} + s_{i-1} - \ldots - s_1 = s_{i+1} - s_1 = \sum_{k=1}^{i} \prod_{l=1}^{k} a_l s_1,$$

yielding

$$s_{i+1} = \sum_{k=1}^{i} \prod_{l=1}^{k} a_l s_1 + s_1, \tag{3}$$

and, since $s_i \to 1$ as $i \to \infty$,

$$s_1 = \frac{1}{1 + \sum\limits_{k=1}^{\infty} \prod\limits_{l=1}^{k} a_l}. \tag{4}$$

With the birth rate satisfying $\lambda_i = \frac{i \cdot \mu_i}{b}$ we have $a_l = \left(\frac{b}{l}\right)$ and therefore

$$\sum_{k=1}^{\infty} \prod_{l=1}^{k} a_l = \sum_{k=1}^{\infty} \frac{b^k}{k!}. \tag{5}$$

This together with Equation (4) gives

$$s_1 = \frac{1}{1 + \sum\limits_{k=1}^{\infty} \frac{b^k}{k!}}, \tag{6}$$

and together with Equation (5) yields the probability of survival from a given state $i > 1$

$$\begin{aligned} s_i &= s_1 + s_1 \sum_{k=1}^{i-1} \frac{b^k}{k!} \\ &= \exp(-b) \sum_{k=0}^{i-1} \frac{b^k}{k!} \\ &= F_{Pois}(i-1; b). \end{aligned}$$

Finally, from the Taylor series

$$1 + \sum_{k=1}^{\infty} \frac{b^k}{k!} = \exp(b)$$

and Equation (6) we obtain the metastasis success probability

$$s_1 = \exp(-b).$$

The assumption of the birth to death ratio satisfying Equation (1) does not define the timing of events in the process. The assumption is enough to derive the probabilities of metastatic success, which, as shown above, do not depend on the particular values of birth λ_i and death rates μ_i.

To describe the timing of this process, a convenient way would be to set

$$\lambda_i = \frac{i}{b},$$

$$\mu_i = 1.$$

In such a way, we could model the real time behavior of the process, where the time unit would be the mean time to death. Thus, realistic time predictions would depend on the measurement of average time to death of cells in a growing metastatic colony.

Consideration of a truncated birth to death rates ratio. We consider a different assumption, namely that

$$\frac{\lambda_i}{\mu_i} = \begin{cases} \frac{i}{b}, & \text{for } i \leq \Delta \\ \frac{\Delta}{b}, & \text{for } i > \Delta, \end{cases}$$

where Δ is a threshold value satisfying $\Delta > b$, and let $\tilde{\Delta} = \lfloor \Delta \rfloor$. We will refer to the stochastic metastasis initiation model with this assumption as the truncated model. The survival probability starting with a single cell in the truncated model is denoted by $s_{1,\Delta}$. Clearly, we have $s_{1,\Delta} < s_1$, where s_1 is the survival probability derived above for the stochastic model without the truncation.

With this assumption, Equations (3) and (4) are also satisfied. Moreover, with a_l defined as above, we obtain

$$a_l = \begin{cases} \frac{b}{l}, & \text{for } l \leq \Delta \\ \frac{b}{\Delta}, & \text{for } l > \Delta, \end{cases}$$

and therefore for the denominator in Equation (4) we get

$$\begin{aligned} 1 + \sum_{k=1}^{\infty} \prod_{l=1}^{k} a_l &= \sum_{k=0}^{\tilde{\Delta}} \frac{b^k}{k!} + \frac{b^{\tilde{\Delta}}}{\tilde{\Delta}!} \sum_{k=\tilde{\Delta}+1}^{\infty} \left(\frac{b}{\Delta}\right)^{k-\tilde{\Delta}} \\ &= e^b \left(1 - \sum_{k=\tilde{\Delta}+1}^{\infty} \frac{b^k}{k!} e^{-b} + e^{-b} \frac{b^{\tilde{\Delta}}}{\tilde{\Delta}!} \cdot \frac{b}{\Delta - b}\right). \end{aligned}$$

Since $s_{1,\Delta} < s_1$, we have $\frac{b^{\tilde{\Delta}}}{\tilde{\Delta}!} \cdot \frac{b}{\Delta - b} > \sum\limits_{k=\tilde{\Delta}+1}^{\infty} \frac{b^k}{k!}$ and hence $s_{1,\Delta}^{-} := e^{-b}\left(1 + e^{-b}\frac{b^{\tilde{\Delta}}}{\tilde{\Delta}!} \cdot \frac{b}{\Delta - b}\right)^{-1}$ is a lower bound to $s_{1,\Delta}$. Defining $c := \frac{\Delta}{b}$ and $\tilde{c} = \frac{\tilde{\Delta}}{b}$ we obtain with the lower bound for the factorial $n! > \sqrt{2\pi} n^{n+\frac{1}{2}} e^{-n}$:

$$\begin{aligned} s_{1,\Delta}^{-} &> e^{-b}\left(1 + e^{-b}\left(\frac{e}{\tilde{c}}\right)^{\tilde{c}b} \cdot \frac{1}{\sqrt{2\pi b} \cdot \tilde{c}\,(c-1)}\right)^{-1} \\ &\geq e^{-b}\left(1 - e^{-b+\tilde{c}b-\tilde{c}b\ln\tilde{c}} \frac{1}{\sqrt{2\pi b} \cdot \tilde{c}\,(c-1)}\right) \\ &\geq e^{-b}\left(1 - e^{-b(1+\tilde{c}(\ln\tilde{c}-1))} \frac{1}{\sqrt{2\pi b} \cdot \tilde{c}\,(c-1)}\right) \end{aligned}$$

Note that $1 + \tilde{c}\,(\ln\tilde{c} - 1) > 0$ for $\tilde{c} > 1$. For $c \geq \tilde{c} \geq e$ we obtain a simple uniform lower bound, namely

$$e^{-b} - e^{-2b} \leq s_{1,\Delta} < e^{-b} = s_1$$

but already for smaller values of c the difference between s_1 and $s_{1,\Delta}$ can essentially be neglected. As an example let $b = 10$ and $c \simeq \tilde{c} = 2$. We obtain

$$e^{-10}\left(1 - 1.9 \times 10^{-3}\right) \leq s_{1,\Delta} < e^{-10} = s_1$$

Hence the error made by replacing the survival probability for the truncated process by the one of the process without truncation is less then two thousand parts in this example.

Generalization of the metastasis initiation model. In the general case, we will consider the following assumption about the ratio of rates λ_i and μ_i for cell division and death, respectively:

$$\frac{\lambda_i}{\mu_i} = \left(\frac{i}{b}\right)^{\alpha}.$$

Here, the exponent α defines the nature of the dependence of proliferation to death rate ratio on colony size. We set $\alpha = 1$, as in the main text, to define linear dependence. Another choice of $\alpha = 1/3$ would correspond to the assumption that the proliferation rate depends on the ratio of the number of cells on the surface to the total number of cells in the colony, assuming spherical metastasis growth (see below). For a population of cells of size i, the individual rates λ_i and μ_i correspond to population proliferation

rate $i \cdot \lambda_i = i^{\alpha+1}/(b)^\alpha \cdot \mu_i$ and population death rate $i \cdot \mu_i$, respectively. We will now derive approximation for the metastasis survival probability in this general case.

Let $\alpha \neq 1$. Following the same argument as above, we can derive

$$s_1 = \frac{1}{1 + \sum_{k=1}^{\infty} \left(\frac{b^k}{k!}\right)^\alpha}. \tag{7}$$

We have:

$$\begin{aligned} \sum_{k=1}^{\infty} \left(\frac{b^k}{k!}\right)^\alpha + 1 &= \exp(\alpha b) \sum_{k=0}^{\infty} \left[\frac{b^k}{k!} \exp(-b)\right]^\alpha \\ &= \exp(\alpha b) \sum_{k=0}^{\infty} \left[p_{Pois}(k, b)\right]^\alpha, \end{aligned}$$

where $p_{Pois}(k, b) = \frac{b^k \exp(-b)}{k!}$ is the Poisson distribution with parameter b.

Jacquet *et al.* [4] showed that for such sequences of distributions $p(k, n)$, which can be approximated with a normal distribution with variance $n\sigma^2 > 0$ for $n \to \infty$, the Renyi entropy satisfies

$$h_n(\alpha) = \frac{1}{1-\alpha} \ln \left[\sum_{k \geq 0} p(k, n)^\alpha\right] = \frac{\ln \left[\alpha^{-\frac{1}{2}} (2\pi n\sigma^2)^{-\frac{\alpha-1}{2}}\right]}{1-\alpha} + o(1).$$

The Poisson distribution with parameter b sufficiently large is well approximated by the the normal distribution with variance b (and mean b). Thus, for the Poisson distribution with rate b its Renyi entropy, denoted $h_{Pois}(b)$, becomes

$$h_{Pois(b)}(\alpha) = \frac{1}{1-\alpha} \ln \left[\sum_{k=0}^{\infty} \left(p_{Pois}(k, b)\right)^\alpha\right] = \frac{\ln \left[\alpha^{-\frac{1}{2}} (2\pi b)^{-\frac{\alpha-1}{2}}\right]}{1-\alpha} + o(1).$$

Simplifying, we obtain

$$\sum_{k=0}^{\infty} \left[p_{Pois}(k, b)\right]^\alpha \approx \alpha^{-\frac{1}{2}} (2\pi b)^{-\frac{\alpha-1}{2}},$$

which leads to

$$\sum_{k=1}^{\infty} \left(\frac{b^k}{k!}\right)^\alpha + 1 = \exp(\alpha b) \sum_{k=0}^{\infty} \left[p_{Pois}(k, b)\right]^\alpha \approx \exp(\alpha b) \alpha^{-\frac{1}{2}} (2\pi b)^{-\frac{\alpha-1}{2}},$$

and after inserting into Equation 7 the metastasis success probability in this model becomes

$$s_1 \approx \alpha^{\frac{1}{2}} \exp(-\alpha b) (2\pi b)^{\frac{\alpha-1}{2}}.$$

Note that for $\alpha = 1$, in agreement with Equation 2, the right hand side equals $\exp(-b)$.

To consider a possible biologically relevant value of $\alpha \neq 1$, we discuss a scenario where the forming colony is a sphere and where, out of all cells in the colony, the cells on the sphere surface are most vulnerable, due to exposure to the surrounding environment. We can further approximate that the total number of cells i is on the order of the volume of the sphere, $i = O(\rho^3)$, and the number of the cells on its surface is $j = O(\rho^2)$, where ρ denotes the radius of the sphere. Thus, $j = O(i^{2/3})$. Assuming that the proliferation rate of cells in the colony depends on the ratio $i/j = i^{1/3}$ between the total to the number of cells on the surface we obtain

$$\lambda_i = \left(\frac{i}{b}\right)^{1/3}.$$

Thus, for spherical metastases, the model parameter is $\alpha = \frac{1}{3}$, and the survival probability becomes

$$s_1 \approx (1/3)^{\frac{1}{2}} (2\pi b)^{-\frac{1}{3}} \exp\left(-\frac{1}{3} b\right).$$

Full derivation of the dependence of metastasis probability on tumor size and bottleneck severity. Denote $t(d)$ the time, measured in years, that elapsed from the onset of the tumor to the time until it got diagnosed with diameter d. Let $N(t)$, for $t \leq t(d)$, be the total number of cells that were released from the primary tumor up to time t and managed to extravasate to the secondary organ. The value of $N(t)$ depends on the primary tumor growth, per-cell per-year release rate, and extravasation probability. We regard the fate of each released tumor cell as independent. Thus, $N(t)$ is interpreted as the number of independent trials that the tumor cells made in order to seed metastases, while $s_1(b)$, defined by the metastasis initiation model, as their success probability. Given that $N(t)$ is very large, while the metastasis success probability $s_1(b)$ is very small, we approximate the distribution of the number of metastatic sites at time t by a Poisson distribution with expectation $s_1(b) \cdot N(t)$. The metastasis probability $M(t;b)$ for time t is then defined as the probability of developing at least one metastatic site up to time t, equal to one minus the probability of having zero metastases under the Poisson distribution

$$M(t;b) = 1 - \exp\left[-s_1(b) \cdot N(t)\right]. \tag{8}$$

Importantly, $M(t;b)$ defines also the cumulative waiting time distribution for the first successful metastasis. Let τ be the random variable describing the waiting time from the onset of the primary tumor to the first metastasis creation. Indeed, the cumulative distribution function for τ equals 1 minus the probability that no metastases have occurred up to time t

$$\Pr\{\tau \leq t\} = 1 - \exp\left[-s_1(b) \cdot N(t)\right] = M(t;b). \tag{9}$$

For a given fixed time point $t^* \leq t(d)$, to compute $N(t^*)$, we consider the per tumor instantaneous rate $Y(t)$ of metastatic cell spread, such that $N(t^*) = \int_0^{t^*} Y(t)\,dt$. The rate $Y(t)$ increases with the tumor volume $X(t)$, measured in cm^3. With additional assumptions of spherical tumor shape and that the majority of cells released from the tumor originate from the tumor surface,

$$Y(t) = u \cdot v \cdot 4\pi \left[\frac{3 \cdot X(t) \cdot 10^9}{4 \cdot \pi}\right]^{2/3}, \tag{10}$$

where the constant u is the per-cell per-year release rate of cells from tumor surface, v is the extravasation probability, $X(t)$ is the volume of the tumor in cm^3 and 10^9 gives the number of cells in unit (1cm^3) volume. To set a realistic release rate u, we assumed that for all cancer types, there are 10^9 cells in a tumor weighting a gram [5], and that around 1.5×10^5 cells are shed from such tumor per day [6], corresponding to around 547.5×10^5 per year. Given that such a tumor of spherical shape has around 4.836×10^6 cells on its surface, we obtained that the per-cell per-year release rate u can be fixed to 11. The extravasation probability v is fixed to 0.8, based on experiments following the fate of tumor cells injected into mice, which stated that around 80% of cells managed to extravasate to secondary organs within three days [7].

To model the tumor volume as a function of time we assume a basic deterministic model of exponential tumor growth [8]

$$X(t) = X_0 \exp(rt),$$

where at time $t = 0$ the tumor volume is $X_0 = 10^{-4}$cm^3, corresponding to 10^5 cells (with volume of a single cell of 10^{-9}cm^3). This initial condition X_0 is chosen to make sure that with X_0, the tumor growth has already passed its early stage of low cell count, in which growth dynamics may be stochastic rather than deterministic. The growth rates r are different for each cancer type and fixed to literature-derived constants (Table **??**). With these assumptions, we can compute the integral

$$N(t^*) = \int_0^{t^*} Y(t)\,dt = u \cdot v \cdot c \cdot (2/3r)^{-1}\left[\exp(2/3rt^*) - 1\right],$$

where the constant $c = (4\pi)^{1/3} \cdot 3^{2/3} \cdot X_0^{2/3} \cdot 10^6$. This fully defines $M(t;b)$ (Equation 8).

With the assumptions of exponential growth for the primary tumor and from the volume expression for its spherical shape we have

$$X_0 \exp\left[r \cdot t(d)\right] = \frac{4\pi}{3}\left(\frac{d}{2}\right)^3,$$

and obtain that a tumor of diameter d has age

$$t(d) = \frac{1}{r}\left[\ln(\pi/6) + 3\ln(d) - \ln(X_0)\right]. \tag{11}$$

To define the metastasis probability as a function of diameter instead of time, we simply use Equation (9) together with (11) and denote

$$M\,(d;b) := M[t(d);b]. \tag{12}$$

Importantly, metastasis probability $M\,(d,b)$ is expected to be much larger than the metastasis detection probability, i.e., incidence of detected metastases in patients with the same tumor size, as it accounts for the probability of having micrometastases, which are not detectable by current screening techniques.

Full derivation of the post-surgical cancer death probability for patients diagnosed with given tumor size. Given the fact that metastases account for circa 90% of cancer deaths, the cancer death probability should depend on the metastasis probability. To make this relationship explicit in the model, we first assume that in order to die of cancer, it is sufficient to have at least one successful metastasis at a distant site. Second, we focus only on patients who had tumors resected following the diagnosis, and assume that the surgery fully removes the source of metastatic seeding. Third, we assume that there is a certain, cancer-specific time δ_0 that on average takes the metastasis to eventually become lethal. Fourth, we also account for the simple fact that patients presenting at diagnosis are alive and their lives were thus not yet threatened by the metastases. The metastasis probability for patients diagnosed with tumor diameter d then becomes a conditional probability, denoted $M(d;b,\delta_0)$, conditioned on the fact that up to time point $t_0(d) = t(d) - \delta_0$, where $t(d)$ is the time of diagnosis with tumor diameter d, no successful metastasis has been created (otherwise the patient would have died prior to diagnosis). This conditional probability can be computed as

$$M\,(d;b,\delta_0) = 1 - \exp\left[-s_1(b)\cdot N\left(t(d);\delta_0\right)\right], \tag{13}$$

where for a given time point t^*

$$N(t^*;\delta_0) = \int_{t_0(d)}^{t^*} Y(t)dt,$$

and $Y(t)$ is the instantaneous per tumor rate of cell spread, given by Equation 10. Computing the integral with the assumption of exponential tumor growth we obtain

$$N(t^*;\delta_0) = u\cdot v\cdot c\cdot (2/3r)^{-1}\left\{\exp\left(2/3rt^*\right) - \exp\left[2/3rt_0(d)\right]\right\},$$

where the constants u, v, and c are defined as above. Fifth, we incorporate effects of treatment in the model, accounting for the fact that post-surgical therapy, most commonly chemotherapy, may eradicate some of metastases that could be life-threatening without treatment. We assume that the metastasis removal is less likely for older, and thus larger metastases, and that the time $t \geq 0$ for the metastases to become irremovable is exponentially distributed with a cancer-specific parameter $1/a$, where a corresponds to the expected age of metastases when they become irremovable. Thus, for metastases which originated t' years before the diagnosis, their probability of being removed be treatment is the probability that they became irremovable at some time point $t > t'$

$$P(t > t') = \exp\left(-\frac{1}{a}t'\right), \tag{14}$$

and decreases with its age t'.

For a given bottleneck severity b, we model cancer death probability as a function of time t counted from tumor onset as the post-treatment, conditional metastasis probability $M(t;a,b,\delta_0)$ that at least one metastasis was successfully initiated by the time t and was not later removed by treatment

$$M(t;a,b,\delta_0) = 1 - \exp\left[-s_1(b)N(t;a,\delta_0)\right]. \tag{15}$$

Here, for a given time point t^*, $N(t^*;a,\delta_0)$ is the number of such metastatic seeding attempts that happened after $t(d) - \delta_0$, up to t^* but also early enough to become irremovable by treatment

$$N(t^*;a,\delta_0) = \int_{t_0(d)}^{t^*}\Big\{1 - \exp\left\{-1/a[t(d) - t]\right\}\Big\}Y(t)dt,$$

where $t(d)$ denotes the time of diagnosis with tumor size d, and the instantaneous per tumor cell spread rate $Y(t)$ (Equation 10) is multiplied by the probability that metastasis, which is $t(d) - t$ years old, is

not removed by treatment after the diagnosis. Solving the integral, we obtain

$$\begin{aligned} N(t^*; a, \delta_0) &= \int_{t_0(d)}^{t^*} Y(t)dt - \exp\left[-(1/a) \cdot t(d)\right] \int_{t_0(d)}^{t^*} \exp\left(1/a \cdot t\right) Y(t)dt \\ &= N(t^*; \delta_0) - \exp\left[-1/a \cdot t(d)\right] \int_{t_0(d)}^{t^*} \exp\left(1/a \cdot t\right) Y(t)dt, \end{aligned}$$

where the integral in the second term can be further simplified to

$$\begin{aligned} \int_{t_0(d)}^{t^*} \exp\left(1/a \cdot t\right) Y(t)dt = \quad & u \cdot v \cdot c \cdot (1/a + 2/3r)^{-1} \\ & \cdot \left\{\exp\left[(1/a + 2/3r)t^*\right] - \exp\left[(1/a + 2/3r)t_0(d)\right]\right\}. \end{aligned}$$

Finally, we model the variability of the bottleneck severity b. Here, we follow the principle that the bottleneck severity, which determines the metastatic success probabilities via $s_1(b) = \exp(-b)$ and is thus an extremely sensitive parameter, should be modeled with a probability distribution and not just by the mean value, as we do with other parameters. From this expression and Equations (8) and (10) it follows, that the bottleneck parameter b enters the rate of the Poisson distribution of the number of metastases in the exponent, while the parameters of the per-cell, per-year release rate of tumor cells u and the extravasation probability v enter only linearly. Hence, the model is much more sensitive to b and less sensitive to u and v. Bottleneck variability is also expected due to biological reasons, such as differences in immune system strengths among patients and between different secondary sites, as well as genetic and phenotypic properties of the tumor seeding cells. The actual values of bottleneck severity b are unknown and with current technology cannot be measured. We assume that b is a random variable from a log normal distribution with density $f(b; \mu, \sigma)$, and cancer-specific parameters location μ and scale σ.

Taking all these considerations together, for patients who underwent tumor surgery followed with therapy reducing metastatic load, their cancer death probability as a function of time from tumor onset is given by the post-treatment, conditional metastasis probability marginalized with respect to the distribution of b

$$M(t; a, \delta_0, \mu, \sigma) = \int_0^{\infty} M(t; a, b, \delta_0) f(b; \mu, \sigma) db. \tag{16}$$

Cancer death probability as a function of tumor diameter d becomes

$$M(d; a, \delta_0, \mu, \sigma) := M[t(d); a, \delta_0, \mu, \sigma]. \tag{17}$$

Thus, the expression for cancer death probability depends on the set of cancer-specific parameters $\{a, \delta_0, \mu, \sigma\}$.

Full derivation of the quantile time to death from cancer for patients with given tumor size. To model any quantile (e.g., median) time it takes to die of cancer, we again make the assumption, that cancer death is due to metastases. We consider the q-th quantile time to death of cancer for patients who (a) had surgery following the diagnosis with diameter d, and (b) will indeed die of cancer. To this end, we need to derive the time $x_q \leq t(d)$ that elapses from tumor onset at which the q-th fraction of patients diagnosed with tumor diameter d develop metastases that were later not removed by post-surgical treatment.

We first consider the conditional cumulative distribution function of x_q, for the waiting time τ for metastases, conditioned on that (i) up to time $t_0(d) = t(d) - \delta_0$ no metastasis has been created, and that (ii) the metastases were present at $t(d)$. For patients with a given value of the bottleneck severity b, the form of such conditional distribution function is

$$\begin{aligned} \Pr\left\{\tau \leq x_q | \tau \geq t_0(d), \tau \leq t(d)\right\} &= \frac{\Pr\left\{\tau \leq x_q, \tau \leq t(d) | \tau \geq t_0(d)\right\}}{\Pr\left\{\tau \leq t(d) | \tau \geq t_0(d)\right\}} \\ &= \frac{\Pr\left\{\tau \leq x_q | \tau \geq t_0(d)\right\}}{\Pr\left\{\tau \leq t(d) | \tau \geq t_0(d)\right\}} \\ &= \frac{M(x_q; b, \delta_0)}{M\left[t(d); b, \delta_0\right]}, \end{aligned}$$

where the last line follows from Equation 9. To account for the variability of the bottleneck, as above we use the marginal probability with respect to the log normal distribution of b with parameters μ and σ. In

addition, we use the post-treatment metastasis probabilities, assuming, again as above, that treatment may remove some of the life-threatening metastases. We thus find x_q as the root of the equation

$$\int_0^\infty \frac{M(x_q; a, b, \delta_0)}{M\left(t(d); a, b, \delta_0\right)} f(b; \mu, \sigma) db = q, \quad t(d) > x_q \geq t_0(d), \tag{18}$$

The quantile time to death is then given by

$$Q\left(d, q; a, \delta_0, h, \mu, \sigma\right) = x_q + \delta_0 - t\left(d\right) + h, \tag{19}$$

where h is an additional treatment-related parameter that accounts for the increase of patient survival due to therapy after detection of the tumor. Thus, the expression for the quantile time to death depends on the cancer-specific parameters $\{a, \delta_0, h, \mu, \sigma\}$, where the dependence on a, μ, and σ is introduced via x_q.

Processing epidemiological data

Patient data was downloaded from the SEER database [9], with the last follow-up date December 31, 2013. We identified 2,091,631 tumor records with given SEER histology and behavior codes that corresponded to one of fourteen common cancer types: ductal and lobular breast, ovarian, endometrial, esophageal, gastric, colon and mucionous colon, rectal, pancreatic, non-small cell lung, head and neck, renal, and bladder cancer. From those, we selected the 549,835 records for adult patients with exactly one tumor of precisely coded diameter below 10 cm at diagnosis, who had surgery but no prior radiation treatment. Tumors were restricted to below 10 cm since there were not enough records for tumors above 10 cm for reliable estimation of modeled variables. To estimate quantile time to death correctly, the patients follow up had to be long enough to result in a representative data distribution. Thus, we defined minimum follow-up times for every cancer type (Table **??**) leading to a selected cohort of 159,191 patients that fulfilled all criteria. From this cohort, different subsets of patient records were defined to estimate different quantities.

To estimate cancer death frequency, we only used records for patients for whom their survival time was recorded, leaving 110,102 records. We grouped these patients by cancer type and tumor diameter on a grid from 0.5 to 10 cm, filtering out groups with less than twenty patients to avoid fluctuations due to small sample sizes. For each group, we aimed at assessing the ratio of patients who died of cancer to the total number of patients in the group. Here, we had to account for the fact that some patients die of reasons other than the tumor disease, and therefore we cannot determine whether they would eventually die of or survive cancer. We thus calculated three estimators of cancer death frequency. For the upper bound estimator, all other deaths are treated as deaths from cancer, while for the lower bound, all dying of other reasons are treated as survivals. The intermediate estimator is computed as 1 minus the Kaplan-Meier estimate of the survival function at its last measured time point, where the survival times of both the other deaths and alive patients contribute to the censored cases.

From the pool of 159,191 patients, metastasis detection frequency was assessed for the subset of 158,358 who had metastasis examined. To this end, for each cancer and tumor diameter group of at least 20 patients, we calculated the fraction of the group who had distant metastases detected at diagnosis.

To extract quantile times to death for all patients, we limited the pre-selected 159,191 patients to 47,166 whose survival time was recorded and who were reported to have died of cancer. For each group of at least twenty patients per each cancer type and each diameter, we calculated their quantile times to death for eleven different quantiles, from the 0.35-th to 0.65-th quantile. For example, the 0.5-th quantile time represents the median time to death, i.e., the number of years for which half of the patient in a group had died of cancer earlier than this number of years.

Finally, we studied a much smaller subset of 9,159 patients, who had metastases examined and the metastases were detected at diagnosis. Again, we evaluated their quantile time to death in cancer- and diameter-resolved groups of at least twenty patients. Here, with the low number of available records, the patient groups were large enough to generate at least two data points for only eight of thirteen cancer types.

Since the SEER database provides only the time of diagnosis, we assumed that diagnosis, surgery, screen for metastatic sites, and tumor measurement took place at the same time point.

Model fitting and validation

Model fitting. The model was fitted to metastasis detection rate and quantile times to death for different tumor diameters, obtained from the SEER database, using nonlinear least squares with the Levenberg-Marquardt algorithm implemented in the minpack.lm R package. The optimization was initialized with a total of 2888 different starting values for the parameters, which were sampled from intervals of sensible values: $\delta_0 \in [0, \inf]$, $\delta_1 \in [0, \inf]$, $h \in [0, \inf]$, $\mu \in [0.1, \inf]$, $\sigma \in [0.001, \inf]$, $a \in [0, 1.09]$, and satisfied the condition $\delta_1 \leq \delta_0$. The upper bound of 1.09 for parameter a corresponds to an upper bound for the probability of removing metastases by treatment (Equation 14). Here, we assume that metastasis which originated 5 years before the diagnosis can be removed with probability at most 1%.

The quantile times to death were computed from the data for eleven different quantiles, from the 0.35-th to 0.65-th quantile. Using quantile time to death data for multiple quantiles (instead only one quantile, e.g. just median) brought power for model fit from a large dataset.

Validation on an independent dataset. For each of the two pancreatic cancer cohorts, Autopsy and Adjuvant, published by Haeno *et al.* [10], we selected patients who had surgery and died of cancer. Next, for each cohort separately, using their clinical data, we computed the survival curves and collected the diameters of patients. For these diameters, we predicted the quantile times to death for quantiles from 0.05 to 0.95 by 0.05, from our model for pancreatic cancer fitted to SEER data, using Equation 19. Finally, to each quantile we mapped the mean predicted time to death for that quantile across the diameters in the cohort. The prediction of the survival function was obtained as one minus the inverse of this mapping. The analysis performed in [10] did not provide the survival functions for the cohorts directly. To obtain the survival function for the model that the authors [10] fit to the Autopsy cohort, we computed cumulative sum of the frequencies predicted by that model for consecutive survival times, read off from Figures 2A,B in [10]. Similarly, the survival function that the same model predicts for the Adjuvant cohort was obtained as cumulative sum of the frequencies plotted in Figures 3B,C,D in [10].

Formulation of the reduced model

The reduced model is considered to show, by comparison to the model introduced above, the importance of capturing bottleneck variability. Accordingly, the reduced model is the same as the above described model and is fitted to data using the same procedure, except that it does not account for bottleneck severity distribution.

Let d denote tumor diameter at diagnosis, $a, b, \delta_0, \delta_1, h$ be the model parameters as defined above, $t(d)$ be the time of diagnosis with tumor diameter d, as defined by Equation (11), and $t_1(d) = t(d) - \delta_1$, where $\delta_1 < \delta_0$. Similarly as above, we model only patients who underwent tumor surgery followed with therapy reducing metastatic load. In the reduced model, for such patients who were diagnosed with a tumor of diameter d, cancer death probability is defined as the post-treatment conditional metastasis probability $M[t(d); a, b, \delta_0]$, defined by Equation (15). Metastasis detection probability is given by $M[t_1(d); b, \delta_0]$, defined by Equation (13). For a given quantile q, the quantile time to death for all patients diagnosed with tumor size d is given by

$$Q\left(d, q; a, b, \delta_0, h\right) = x_q + \delta_0 - t\left(d\right) + h, \tag{20}$$

where x_q is the root of

$$\frac{M(x_q; a, b, \delta_0)}{M\left(t(d); a, b, \delta_0\right)} = q, \quad t(d) > x_q \geq t_0(d). \tag{21}$$

Finally, the quantile q time to death for the subset of patients with metastases detected at diagnosis with a tumor of given size d is given by

$$Q'\left(d, q; a, b, \delta_0, h\right) = x'_q + \delta_0 - t\left(d\right) + h, \tag{22}$$

where x'_q is the root of the equation

$$\frac{M(x'_q; a, b, \delta_0)}{M\left(t_1(d); a, b, \delta_0\right)} = q, \quad t_1(d) > x'_q \geq t_0(d). \tag{23}$$

References

[1] Cisneros, L. H. & Newman, T. J. Quantifying metastatic inefficiency: rare genotypes versus rare dynamics. *Phys Biol* **11**, 046003 (2014).

[2] Athreya, K. & Ney, P. *Branching processes* (Dover, Mineola, New York, 1972).

[3] Haccou, P., Jagers, P. & Vatutin, V. A. (eds.) *Branching processes: Variation, growth, and extinction of populations* (Cambridge University Press, 2005).

[4] Jacquet, P., Szpankowski, W. & N, L. Entropy computations via analytic depoissonization. *IEEE Trans. Information Theory* **45**, 1072–1081 (1998).

[5] DeVita, V. T., Young, R. C. & Canellos, G. P. Combination versus single agent chemotherapy: a review of the basis for selection of drug treatment of cancer. *Cancer* **35**, 98–110 (1975).

[6] Liotta, L. A., Kleinerman, J. & Saidel, G. M. Quantitative relationships of intravascular tumor cells, tumor vessels, and pulmonary metastases following tumor implantation. *Cancer Res.* **34**, 997–1004 (1974).

[7] Luzzi, K. J. Multistep nature of metastatic inefficiency: dormancy of solitary cells after successful extravasation and limited survival of early micrometastases. *Am. J. Pathol.* **153**, 865–873 (1998).

[8] Gerlee, P. The model muddle: in search of tumour growth laws. *Cancer Research* (2013).

[9] SEER. Surveillance, Epidemiology, and End Results Program (www.seer.cancer.gov) Research Data (1973-2013), National Cancer Institute, DCCPS, Surveillance Research Program, Surveillance Systems Branch (released in Nov 2015).

[10] Haeno, H. *et al.* Computational modeling of pancreatic cancer reveals kinetics of metastasis suggesting optimum treatment strategies. *Cell* **148**, 362 – 375 (2012).
