# Supplementary material for: A mathematical model of the metastatic bottleneck predicts patient outcome and response to cancer treatment
Source: PLoS Comput Biol. 2020 Oct 2;16(10):e1008056. doi: 10.1371/journal.pcbi.1008056 (PMC7591057; doi:10.1371/journal.pcbi.1008056)
Supplement: S1 Table — For each cancer type, we list the tumor doubling time in days, denoted D, as found reported (usually as median or mean value across a measured population) in the cited references. The per year exponential growth rate r in our model, was then computed from those doubling times as ln(2) ⋅ 365.24/D. For several cancer types, we were unable to find measured doubling times. For those cancer types, we fixed the corresponding rates to ones measured in the most similar cancer type. Specifically, for endometrial cancer, we fixed the doubling rate to the rate reported in another female cancer, breast. For esophageal, gastric and bladder cancer types, we fixed the rate as reported for colon and rectum. (PDF) [file pcbi.1008056.s011.pdf]

Table S1. **Per-cancer tumor growth rates fixed for thirteen modeled cancer types.**

Cancer	Doubling time D (days)	Reference
Breast	212	[?]
Breast lob	212	[?]
Endometrial	212	[?]
Esophageal	255	[?]
Gastric	255	[?]
Colon	255	[?]
Colon muc	255	[?]
Rectal	255	[?]
Pancreatic	144	[?]
Lung	166,3	[?]
Head & neck	99	[?]
Renal	603	[?]
Bladder	255	[?]

References

[1] von Fournier, D. *et al.* Growth rate of 147 mammary carcinomas. *Cancer* **45**, 2198–2207 (1980).

[2] Choi, S. J., Kim, H. S., Ahn, S. J., Jeong, Y. M. & Choi, H. Y. Evaluation of the growth pattern of carcinoma of colon and rectum by MDCT. *Acta Radiol* **54**, 487–492 (2013).

[3] Furukawa, H., Iwata, R. & Moriyama, N. Growth rate of pancreatic adenocarcinoma: initial clinical experience. *Pancreas* **22**, 366–369 (2001).

[4] Arai, T. *et al.* Tumor doubling time and prognosis in lung cancer patients: evaluation from chest films and clinical follow-up study. Japanese Lung Cancer Screening Research Group. *Jpn. J. Clin. Oncol.* **24**, 199–204 (1994).

[5] Jensen, A. R., Nellemann, H. M. & Overgaard, J. Tumor progression in waiting time for radiotherapy in head and neck cancer. *Radiother Oncol* **84**, 5–10 (2007).

[6] Ozono, S. *et al.* Tumor doubling time of renal cell carcinoma measured by CT: collaboration of Japanese Society of Renal Cancer. *Jpn. J. Clin. Oncol.* **34**, 82–85 (2004).
