# Supplementary material for: A mathematical model of the metastatic bottleneck predicts patient outcome and response to cancer treatment
Source: PLoS Comput Biol. 2020 Oct 2;16(10):e1008056. doi: 10.1371/journal.pcbi.1008056 (PMC7591057; doi:10.1371/journal.pcbi.1008056)
Supplement: S2 Table — For each cancer type, we provide a short name that is used throughout the manuscript. For each type, we defined a designated minimum follow-up time that ensures a sufficient number of patients for calculating representative statistics of frequency of metastasis detection, frequency of cancer death and quantile times to death. Generally, the minimum follow up times were a compromise between the requirement of long monitoring time after diagnosis, and the resulting sample sizes for each cancer. The minimum follow-up time differs between different cancer types, reflecting their clinical behavior. For example, for more aggressive cancers, such as pancreatic and esophageal cancer, which have a poor prognosis, a shorter minimum follow-up time was allowed. In contrast, for breast cancer, where the ten year survival rate for all stages is nearly 80%, the minimum follow-up was set to 20 years. This still allowed for a very large sample size of 35949 patients, since breast cancer is one of the most common cancer types. (PDF) [file pcbi.1008056.s012.pdf]

Table S2. **Overview of analyzed cancer types with minimum follow up times and number of cases included after filtering from the SEER database.**

Cancer type short name	Cancer type	Min. follow up in years	Number of cases included
Breast	Invasive ductal carcinoma	20	35949
Breast lob	Invasive lobular carcinoma	15	6532
Ovarian	Epithelial ovarian cancer	15	2370
Endometrial	Endometrial carcinoma	15	6941
Esophageal	Esophageal carcinoma	10	1725
Gastric	Gastric adenocarcinoma	10	8238
Colon	Adenocarcinoma of the colon (except mucinous)	15	45538
Colon muc	Mucinous adenocarcinoma of the colon	15	3739
Rectal	Adenocarcinoma of the rectum	15	8334
Pancreatic	Exocrine pancreatic cancer	10	2618
Lung	Non-Small cell lung cancer	15	18587
Head & neck	Head and neck squamous-cell carcinoma (HNSCC)	15	8046
Renal	Renal cell carcinoma	15	8133
Bladder	Urothelial carcinoma	15	2441
