# Supplementary material for: A mathematical model of the metastatic bottleneck predicts patient outcome and response to cancer treatment
Source: PLoS Comput Biol. 2020 Oct 2;16(10):e1008056. doi: 10.1371/journal.pcbi.1008056 (PMC7591057; doi:10.1371/journal.pcbi.1008056)
Supplement: S3 Table — For each cancer type (rows), the p-values in a two-sided Mann-Kendall trend test are reported. Table entries in red contain p-values lower than 0.01. Light red entries contain p-values lower than 0.05. For metastasis detection probability, cancer death probability and median time to death, the monotonic trend is significant for all cancers, except ovarian and with exception of cancer death probability for esophageal and median time to death for colon mucinous cancer. The same results hold for false discovery rate (FDR) computed from p-values using Benjamini-Hochberg correction for 14 tests (first four columns) or without correction (last four columns). NA values in the “Median time to death for patients with mets” column indicate that, due to too small patient sample sizes, there is no such data available. (PDF) [file pcbi.1008056.s013.pdf]

Table S3. **Significance analysis of the monotonic dependence of clinical variables on tumor size.**

	FDR (Benjamini-Hochberg adjusted p-values)				p-values			
Cancer	Met detection prob.	Cancer death prob.	Median time to death	Median time to death with mets	Met detection prob.	Cancer death prob.	Median time to death	Median time to death with mets
Breast	8.19e-07	3.84e-06	3.21e-06	1.54e-01	3.28e-08	6.91e-07	3.85e-07	1.33e-01
Breast lob	1.15e-05	6.74e-05	5.40e-04	NA	3.23e-06	3.09e-05	3.03e-04	NA
Ovarian	2.56e-01	3.81e-02	7.07e-01	4.86e-01	2.30e-01	2.97e-02	6.78e-01	4.48e-01
Endometrial	7.70e-06	7.88e-05	6.30e-04	NA	1.85e-06	3.78e-05	3.66e-04	NA
Esophageal	6.74e-05	5.81e-01	6.63e-04	NA	3.10e-05	5.46e-01	4.01e-04	NA
Gastric	7.17e-06	1.51e-05	1.51e-05	7.34e-01	1.58e-06	5.12e-06	4.89e-06	7.19e-01
Colon	5.82e-06	3.84e-06	6.04e-07	1.49e-02	1.16e-06	6.28e-07	1.21e-08	1.10e-02
Colon muc	5.08e-04	4.24e-02	7.85e-01	2.02e-01	2.74e-04	3.39e-02	7.85e-01	1.78e-01
Rectum	3.84e-06	1.44e-06	1.40e-05	1.36e-01	6.28e-07	8.64e-08	4.19e-06	1.14e-01
Pancreas	1.60e-05	2.57e-02	1.19e-04	NA	5.76e-06	1.95e-02	5.96e-05	NA
Lung	5.00e-03	6.63e-04	3.21e-06	1.03e-02	3.40e-03	4.11e-04	3.19e-07	7.43e-03
Head & neck	1.75e-04	3.15e-05	8.46e-06	NA	9.10e-05	1.26e-05	2.20e-06	NA
Renal	3.21e-06	6.40e-05	5.63e-03	8.72e-02	3.26e-07	2.69e-05	3.94e-03	7.15e-02
Bladder	2.68e-05	1.65e-03	9.20e-04	NA	1.02e-05	1.09e-03	5.89e-04	NA
